# Supplementary material for: mRNA delivery enabled by metal–organic nanoparticles
Source: Nat Commun. 2024 Nov 8;15:9664. doi: 10.1038/s41467-024-53969-w (PMC11544223; doi:10.1038/s41467-024-53969-w)
Supplement: Supplementary file 1 — Supplementary Information [file 41467_2024_53969_MOESM1_ESM.pdf]

# Supplementary Information

## mRNA Delivery Enabled by Metal-Organic Nanoparticles

*Yuang Gu[1‡], Jingqu Chen[1‡], Zhaoran Wang[1], Chang Liu[1], Tianzheng Wang[1], Chan-Jin Kim[1], Helena Durikova[1], Soraia Fernandes[1], Darryl N. Johnson[2], Robert De Rose[1], Christina Cortez-Jugo[1], & Frank Caruso[1,*]*

[1]Department of Chemical Engineering, The University of Melbourne, Parkville, Victoria 3010, Australia

[2]Materials Characterisation and Fabrication Platform, The University of Melbourne, Parkville, Victoria 3010, Australia

*e-mail: fcaruso@unimelb.edu.au

‡These authors contributed equally to this work

.

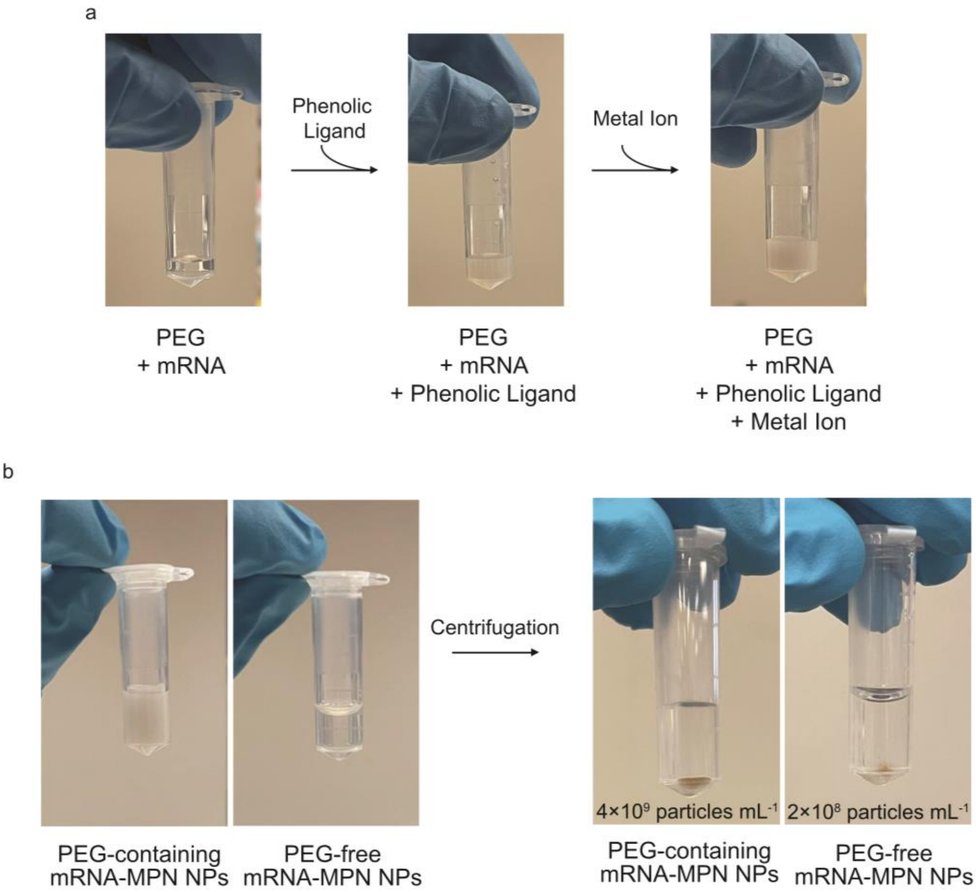


**Supplementary Fig. 1 | a,** Photographs showing the assembly process of mRNA-MPN NPs (assembled with 20k linear PEG, mCherry, EGCG, and $Zr^{IV}$ at a mass ratio of 100:1:100:2.5). Upon introduction of the phenolic ligand, EGCG, the turbidity of the solution increased, indicating the occurrence of cross-linking between PEG and EGCG. Within a few seconds of adding the metal ions, $Zr^{IV}$, the mixture transitioned to a milky white color, suggesting complexation between $Zr^{IV}$ and EGCG. **b,** Yield of mRNA-MPN NPs prepared with or without the inclusion of PEG. The turbidity of the NP suspension provided a qualitative indication of NP concentration while the number of mRNA-MPN NPs was quantified by NanoSight N300 (Malvern Panalytical, UK).

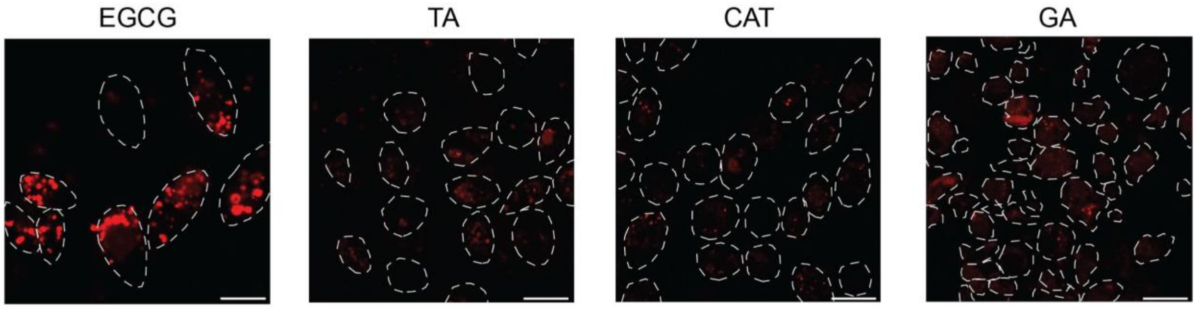


**Supplementary Fig. 2 |** Representative CLSM images showing HEK 293T cells transfected by mRNA-MPN NPs after 24 h. The NPs were assembled with different phenolic ligands (EGCG, TA, CAT, or GA) and the mass ratio of the NP formulation examined was 20k linear PEG:mRNA:phenolic ligands:$Zr^{IV}$ = 100:1:100:2.5. Cell membranes are indicated by the dotted lines. Scale bars are 20 μm. All experiments were performed in triplicates.

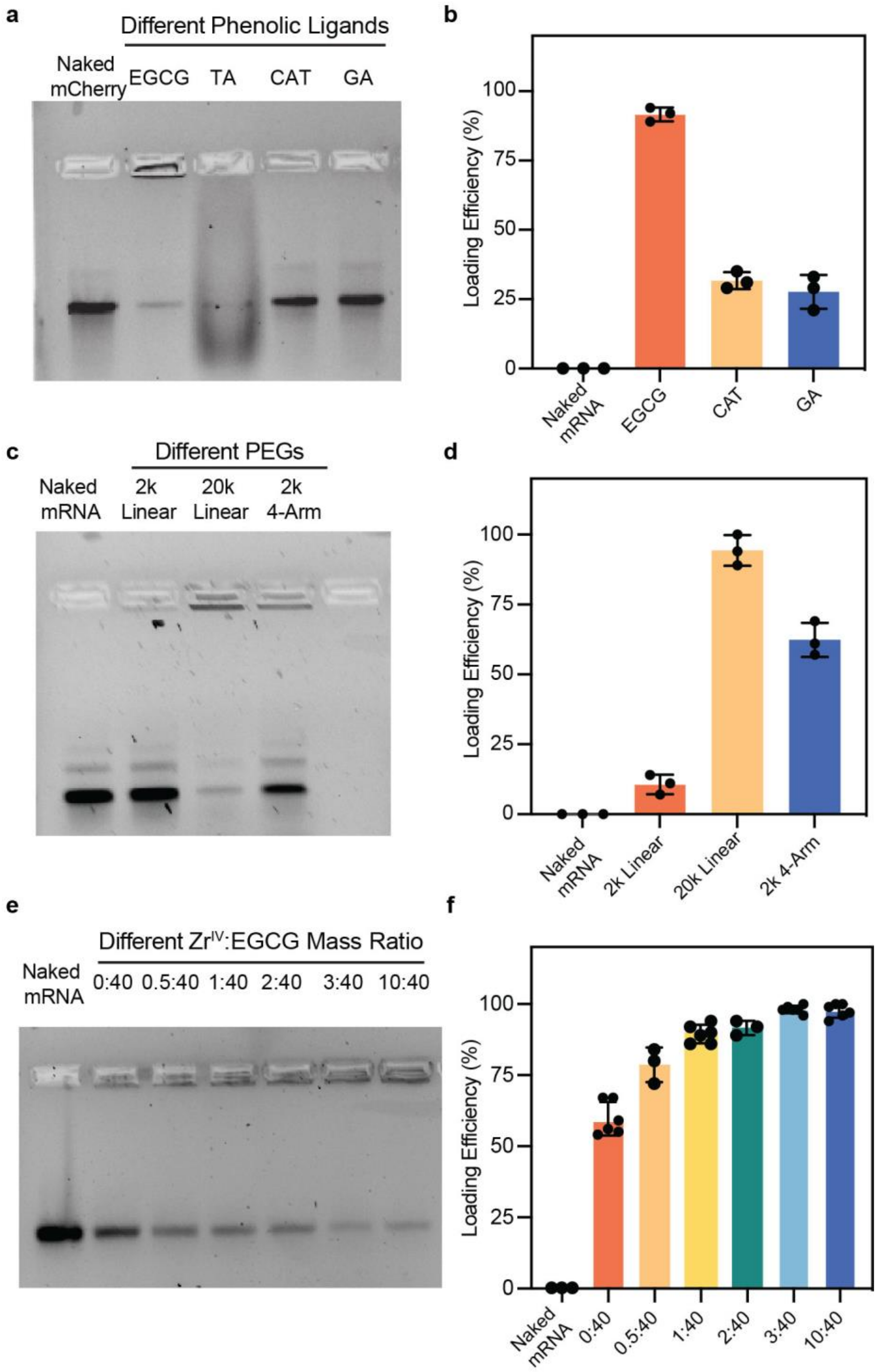


**Supplementary Fig. 3 |** Loading of mCherry into different NP formulations, as assessed by agarose gel electrophoresis. **a,b,** Qualitative (**a**) and quantitative (**b**) loading analyses using formulations prepared with different phenolic ligands; the mass ratio of the NP formulations was 20k linear PEG:mRNA:phenolic ligands:$Zr^{IV}$ = 100:1:100:2.5. **c,d,** Qualitative (**c**) and quantitative (**d**) loading analyses using formulations prepared by varying the $M_w$ or structure of PEG; the mass ratio of the NP formulations was PEG:mRNA:EGCG:$Zr^{IV}$ = 100:1:100:2.5. **e,f,** Qualitative (**e**) and quantitative (**f**) loading analyses using formulations comprising 20k linear PEG, mRNA, EGCG, and $Zr^{IV}$ at different $Zr^{IV}$-to-EGCG mass ratios. All experiments were performed in triplicates or hexaplicates ($n$ = 3 or 6) and data are presented as the

mean ± SD. Purification of the NPs was not carried out for these experiments and the results related to the TA-based NPs are not shown in (b) because of the development of a smeared band in the gel. This is likely due to the interaction of TA with the gel-loading dye. Note that the RiboGreen assay was not used for loading quantification owing to incompatibility of our particle constituents with the assay kit, as determined from the product manual (Thermo Fisher Scientific, R11490). Source data are provided as a Source Data file.

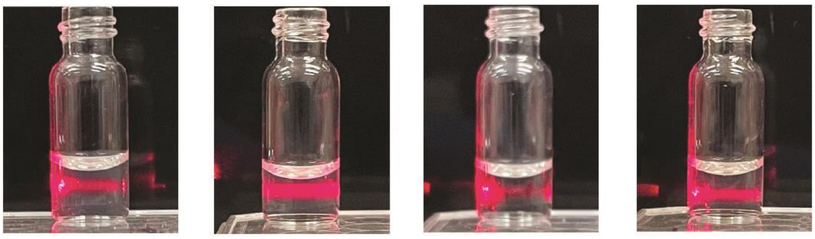


**Supplementary Fig. 4 |** Photographs showing Tyndall effects in mRNA-MPN NP dispersions assembled with different phenolic ligands. The NP formulations were prepared using 20k linear PEG, mCherry, phenolic ligand, and $Zr^{IV}$ at a mass ratio of 100:1:100:2.5. The scattering of the light beam in the solution of EGCG-, TA-, and GA-incorporated NPs is indicative of successful colloidal particle formation.

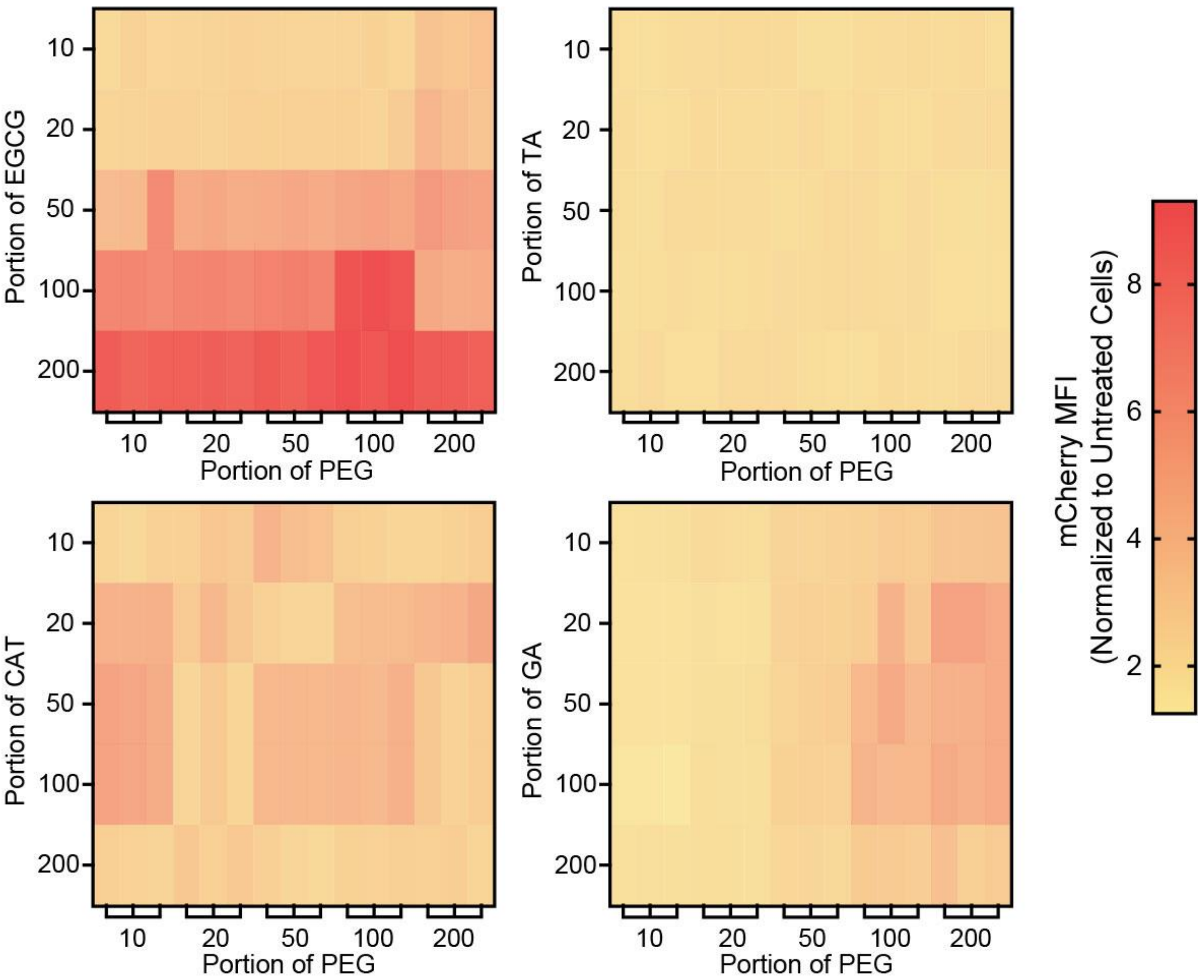


**Supplementary Fig. 5 |** Heat map showing the MFI of mCherry following transfection of HEK 293T cells using mRNA-MPN NPs assembled with different phenolic ligands at a dosage of 100 ng per well in 96-well plates. The NPs were synthesized using fixed amounts of mCherry mRNA (100 ng, set as 1 portion) and $Zr^{IV}$ (250 ng, 2.5 portion) and varying mass portions of phenolic ligands and 20k linear PEG. All experiments were conducted in triplicates. Source data are provided as a Source Data file.

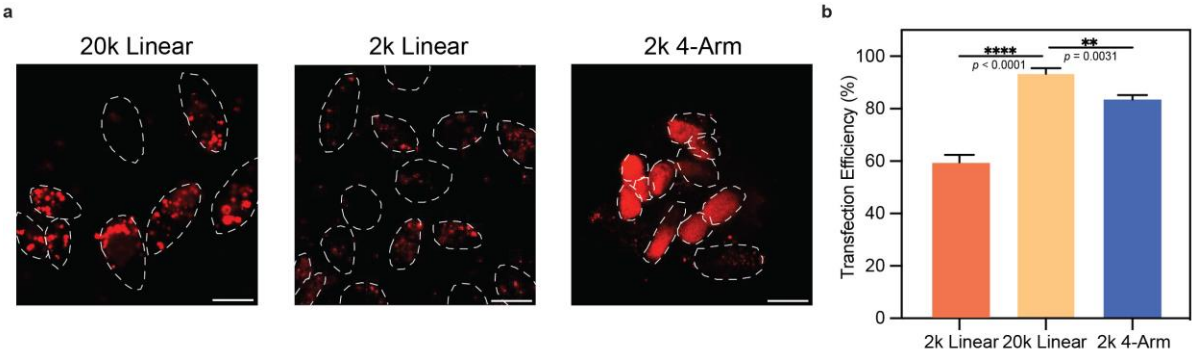


**Supplementary Fig. 6 | a,** Representative CLSM images showing HEK 293T cells transfected with mRNA-MPN NPs after 24 h. The NPs were assembled by varying the $M_w$ or structure of PEG (the mass ratio of the NP formulations was PEG:mRNA:EGCG:$Zr^{IV}$ = 100:1:100:2.5). Cell membranes are indicated by the dotted lines. Scale bars are 20 µm. **b,** Percentage of mCherry$^+$ HEK 293T cells transfected by the corresponding NP formulations after 24 h. All experiments were performed in triplicates ($n = 3$) and data are presented as the mean ± SD. Statistical significance was analyzed using one-way ANOVA: ****$p < 0.0001$; ***$p < 0.001$; **$p < 0.01$; *$p < 0.05$; ns, $p > 0.05$ with Tukey's multiple comparisons test. $p$ (2k Linear vs 20k Linear) = $3.9 \times 10^{-6}$, $p$ (20k Linear vs 2k 4-Arm) = 0.0031. Source data are provided as a Source Data file.

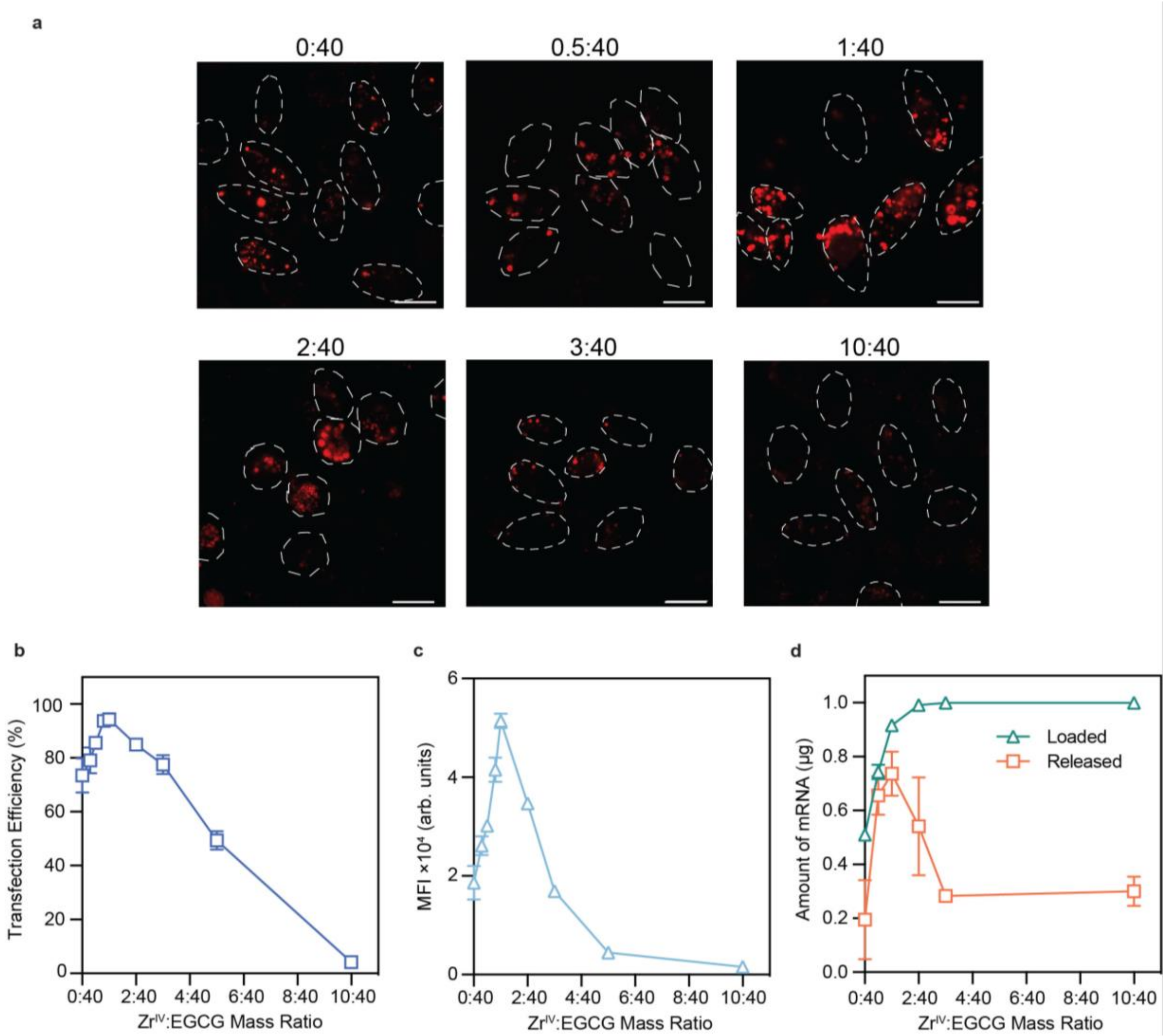


**Supplementary Fig. 7 | a,** Representative CLSM images of HEK 293T cells transfected by mRNA-MPN NPs after 24 h. The NP formulations comprising 20k linear PEG, EGCG, and $Zr^{IV}$ were prepared using different metal ion concentrations (effectively $Zr^{IV}$-to-EGCG mass ratios). Cell membranes are indicated by the dotted lines. Scale bars are 20 μm. **b,c,** Percentage of $mCherry^{+}$ HEK 293T cells (**b**) and mCherry MFI of HEK 293T (**c**) transfected by the corresponding formulations after 24 h. **d,** mRNA loading and release corresponding to different $Zr^{IV}$-to-EGCG mass ratios. All experiments were performed in triplicates or pentaplicates ($n$ = 3 or 5) and data are presented as the mean ± SD. Source data are provided as a Source Data file.

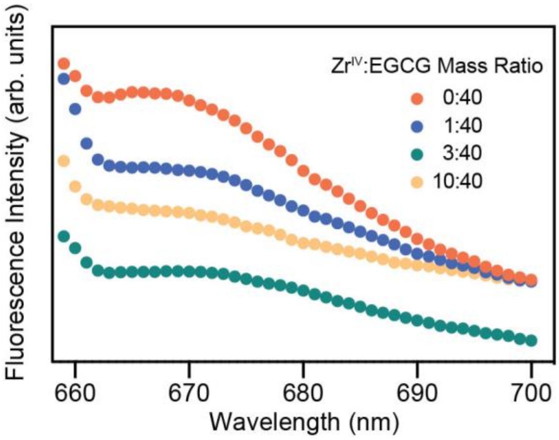

**Supplementary Fig. 8 |** Influence of metal ion concentration on fluorescence quenching of Cy5-labeled mRNA as a representative fluorescent source. The NP formulations comprising 20k linear PEG, mRNA, EGCG, and $Zr^{IV}$ were prepared with different $Zr^{IV}$-to-EGCG mass ratios. Source data are provided as a Source Data file.

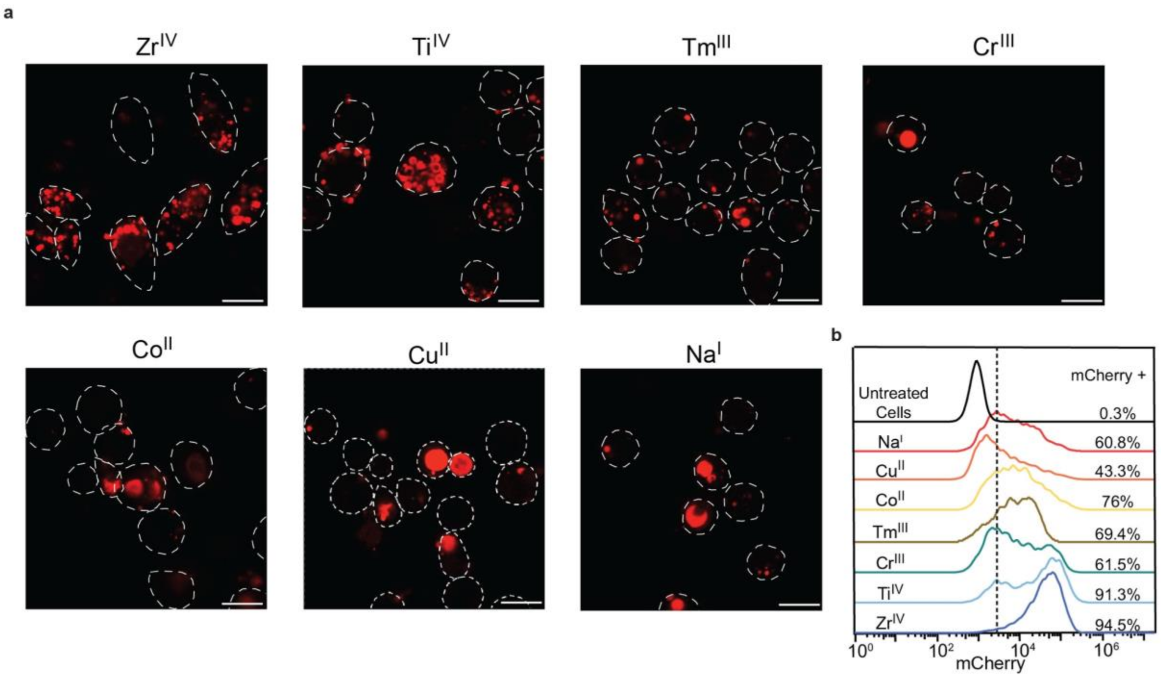


**Supplementary Fig. 9 | a,** Representative CLSM images showing HEK 293T cells transfected by mRNA-MPN NPs after 24 h. The NPs were assembled with different metal ions (the mass ratio of the NP formulations was 20k linear PEG:mRNA:EGCG:metal ion = 100:1:100:2.5). Cell membranes are indicated by the dotted lines. Scale bars are 20 μm. **b,** Histogram of mCherry fluorescence of HEK 293T cells transfected by the corresponding formulations. All experiments were performed in triplicates ($n$ = 3).

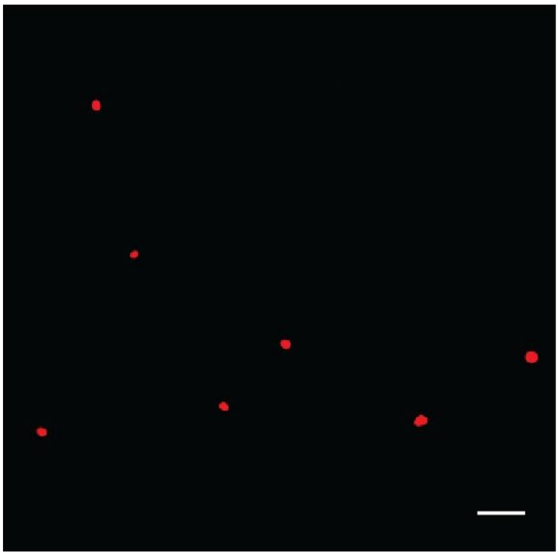

**Supplementary Fig. 10 |** Representative Lattice-SIM image showing the hydrous morphology of mRNA-MPN NPs (lead formulation). The NPs were prepared using 20k linear PEG, mRNA, EGCG, metal ion at a mass ratio of 100:1:100:2.5. mRNA was labeled with Cy5 (red). Scale bar is 1 µm. All experiments were performed in triplicates.

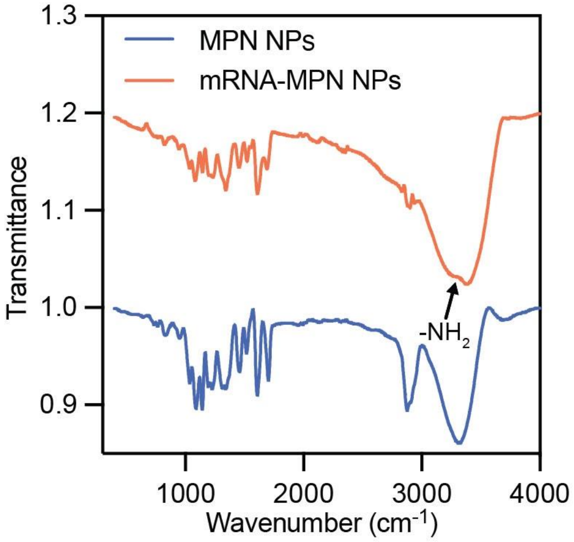


**Supplementary Fig. 11 |** FTIR spectra of mRNA-MPN NPs (lead formulation) and complex of EGCG, PEG, and $Zr^{IV}$. Source data are provided as a Source Data file.

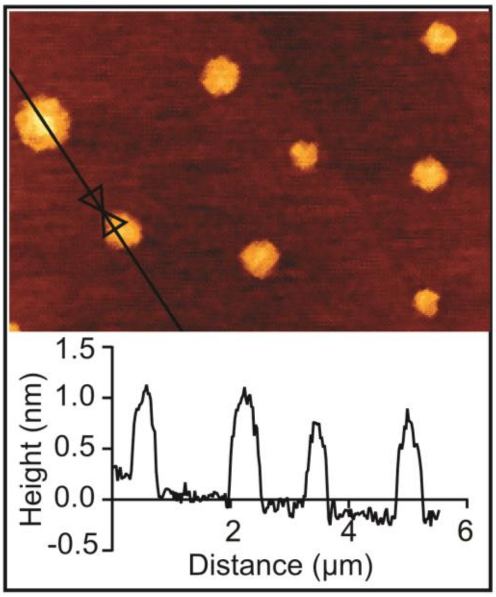


**Supplementary Fig. 12 |** AFM image and corresponding thickness profile of mRNA-MPN NPs in the air-dried state. Source data are provided as a Source Data file.

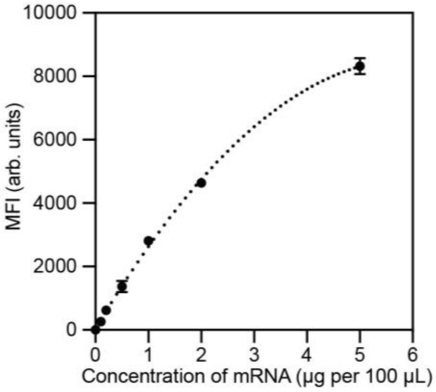


**Supplementary Fig.13 |** Fluorescence intensity of Cy5-labeled mRNA as a function of mRNA concentration. Experiments were performed in technical triplicates (*n* = 3) and data are presented as the mean ± SD. Source data are provided as a Source Data file.

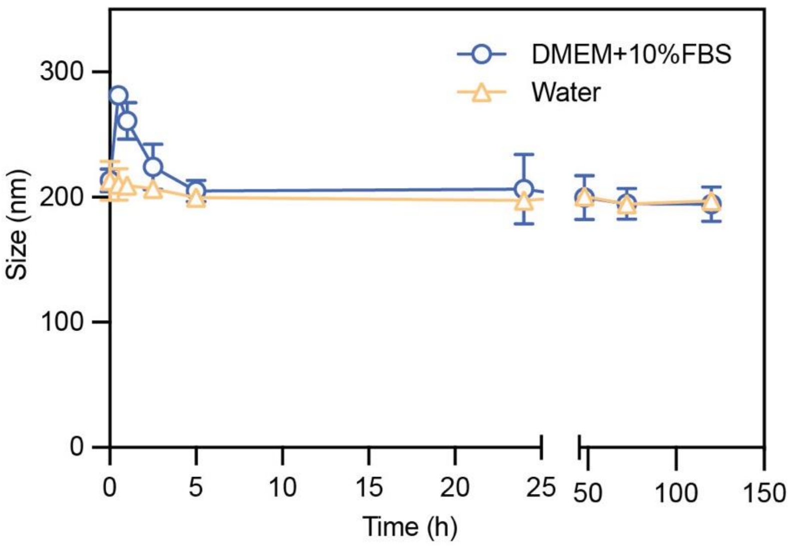


**Supplementary Fig.14 |** Change in size of mRNA-MPN NPs in water and DMEM+10% FBS over 120 h. Experiments were performed in triplicates (*n* = 3) and data are presented as the mean ± SD. Source data are provided as a Source Data file.

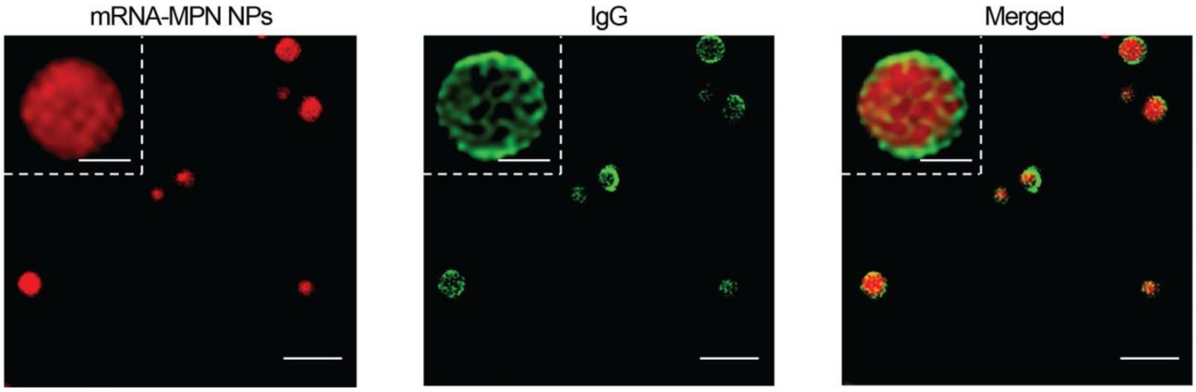


**Supplementary Fig. 15 |** Lattice-SIM images of mRNA-MPN NPs following adsorption of antibodies. mRNA (red) was conjugated with Cy5 and IgG (green) was conjugated with AF488. Scale bars in the main images are 1 µm. The insets are magnified images of representative particles; scale bars are 200 nm. Experiments were performed in triplicates.

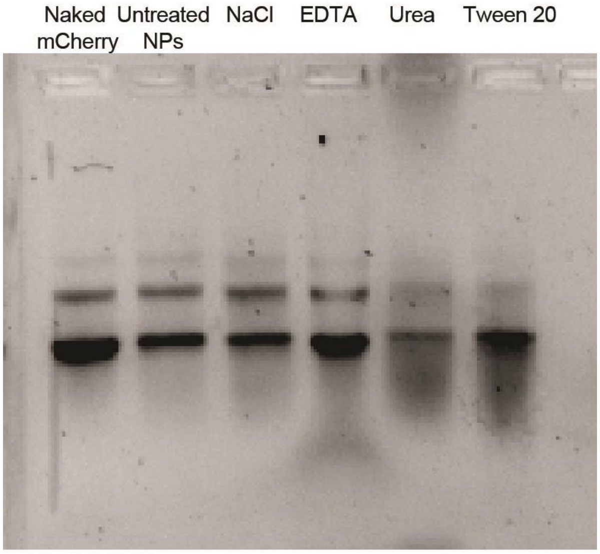


**Supplementary Fig. 16 |** Representative agarose gel bands showing mRNA release pattern after incubation of mRNA-MPN NPs for 24 h in different solutions.

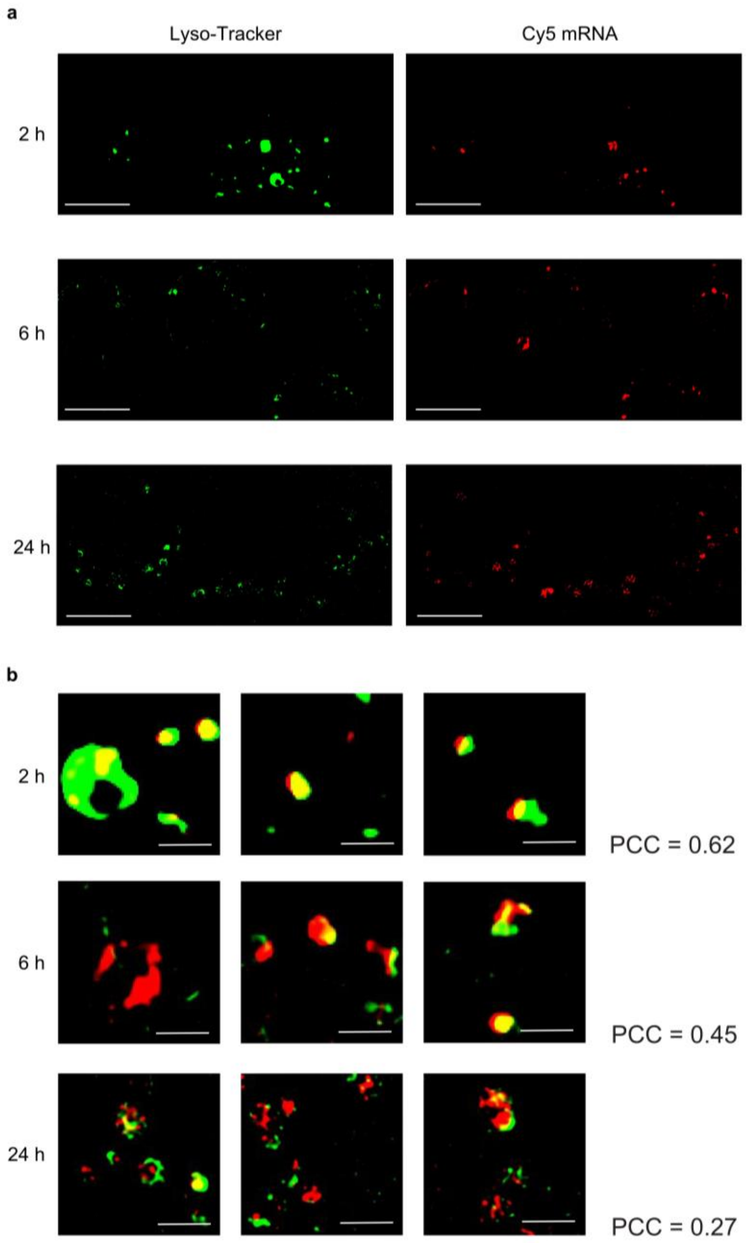


**Supplementary Fig. 17 | a,** Representative SIM images of the intracellular colocalization of mRNA-MPN NPs with endo/lysosomes 2,6, and 24 h post incubation. Endo/lysosomes (green) were stained with LysoTracker Green DND-26 and mRNA (red) was conjugated with Cy5. NP formulation: 20k linear PEG, mRNA, EGCG, and $Zr^{IV}$ at a mass ratio of 100:1:100:2.5. Scale bars are 5 µm. **b,** Representative high-magnification images. Scale bars are 2 µm. PCC, Pearson's correlation coefficient. Experiments were performed in triplicates.

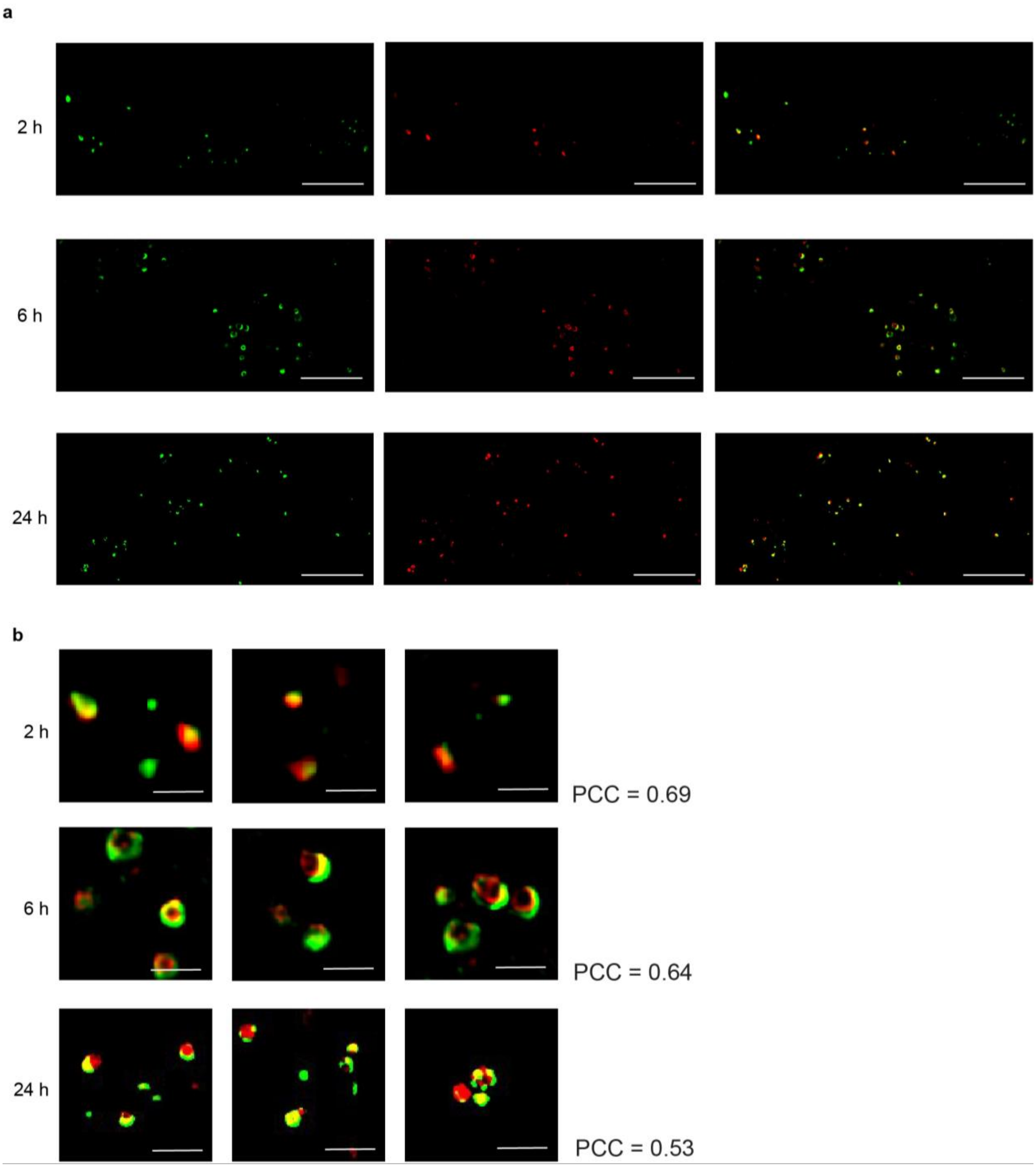


**Supplementary Fig. 18 | a,** Representative SIM images of the intracellular colocalization of the metal ion-free formulation with endo/lysosomes 2, 6, and 24 h post incubation. Endo/lysosomes (green) were stained with LysoTracker Green DND-26 and mRNA (red) was conjugated with Cy5. NP formulation: 20k linear PEG, mRNA, and EGCG at a mass ratio of 100:1:100. Scale bars are 5 µm. **b,** Representative high-magnification images. Scale bars are 2 µm. Experiments were performed in triplicates.

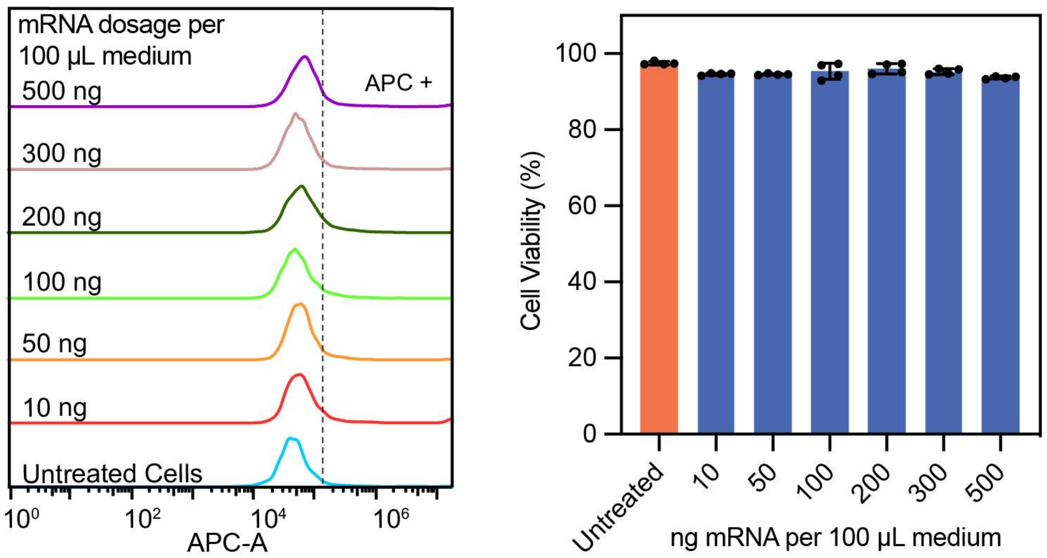


**Supplementary Fig. 19 |** Viability of HEK 293T cells post treatment with different concentrations of mRNA-MPN NPs. All experiments were performed in quadruplicates (*n* = 4) and data are presented as the mean ± SD. Source data are provided as a Source Data file.

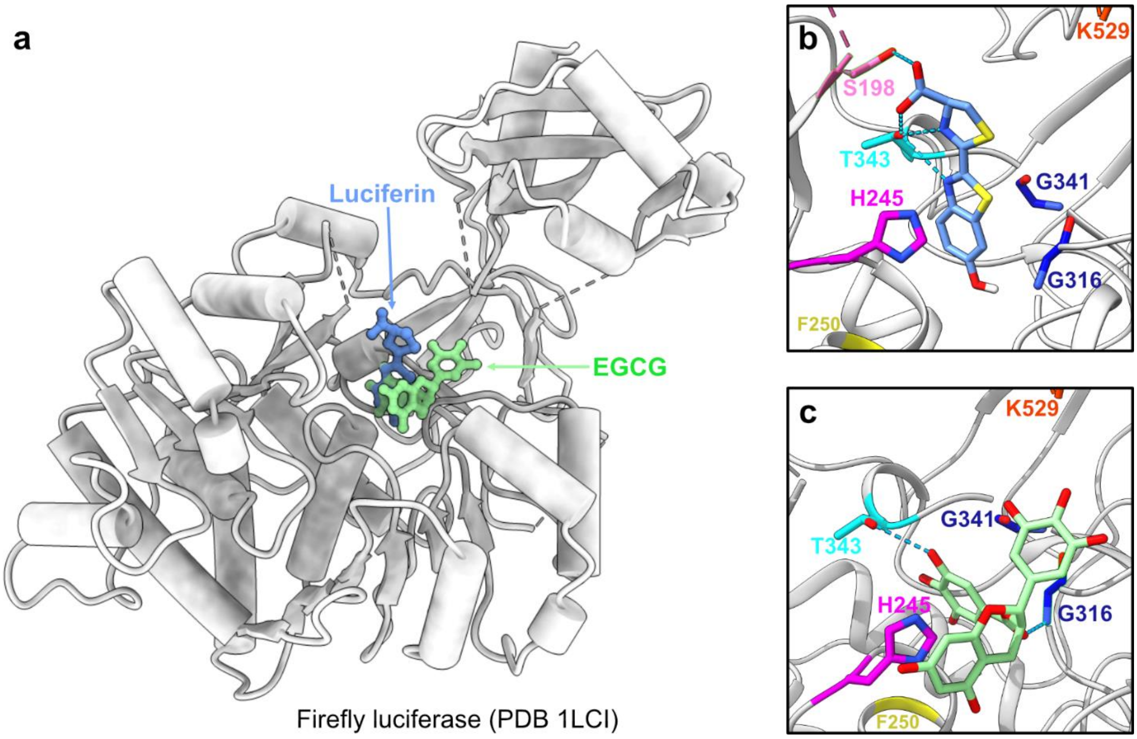


**Supplementary Fig. 20 |** Competitive binding of EGCG at binding sites of luciferin simulated using Autodock Vina. **a,** Luciferin and EGCG superpositioned on firefly luciferase (FLuc) (PDB ID 1LCI). Grey, protein backbone; blue, luciferin (PubChem CID 92934); green, EGCG (PubChem CID 65064). **b**,**c**, Comparison of proximate residual interactions between FLuc and EGCG in the common binding pocket. To investigate the unexpected quenching effect of MPN NPs on the activity of luciferase, we examined the spatial interactions between EGCG and FLuc. We computationally docked FLuc (PubChem CID 92934) and EGCG (PubChem CID 65064) with Autodock Vina and successfully docked luciferin to firefly luciferase (**b**) with the lowest predicted affinity of −6.319 kcal $mol^{-1}$. FLuc are coordinated by S198 and T343 through hydrogen bonds, in line with the experimentally verified active site[1,2]. The superposition of luciferin and EGCG (docked at the same grid) revealed a significant spatial overlap at the active site. Further structural analysis suggested that EGCG were coordinated by T343 and G316 by hydrogen bonds and stabilized by possible π-π stacking between H245 and the phenylic group. EGCG was docked at the pocket with minimal affinity of −7.58 kcal $mol^{-1}$. We therefore rationalize that free EGCG may act as an inhibitor to luciferase, possibly through direct or combined competition.

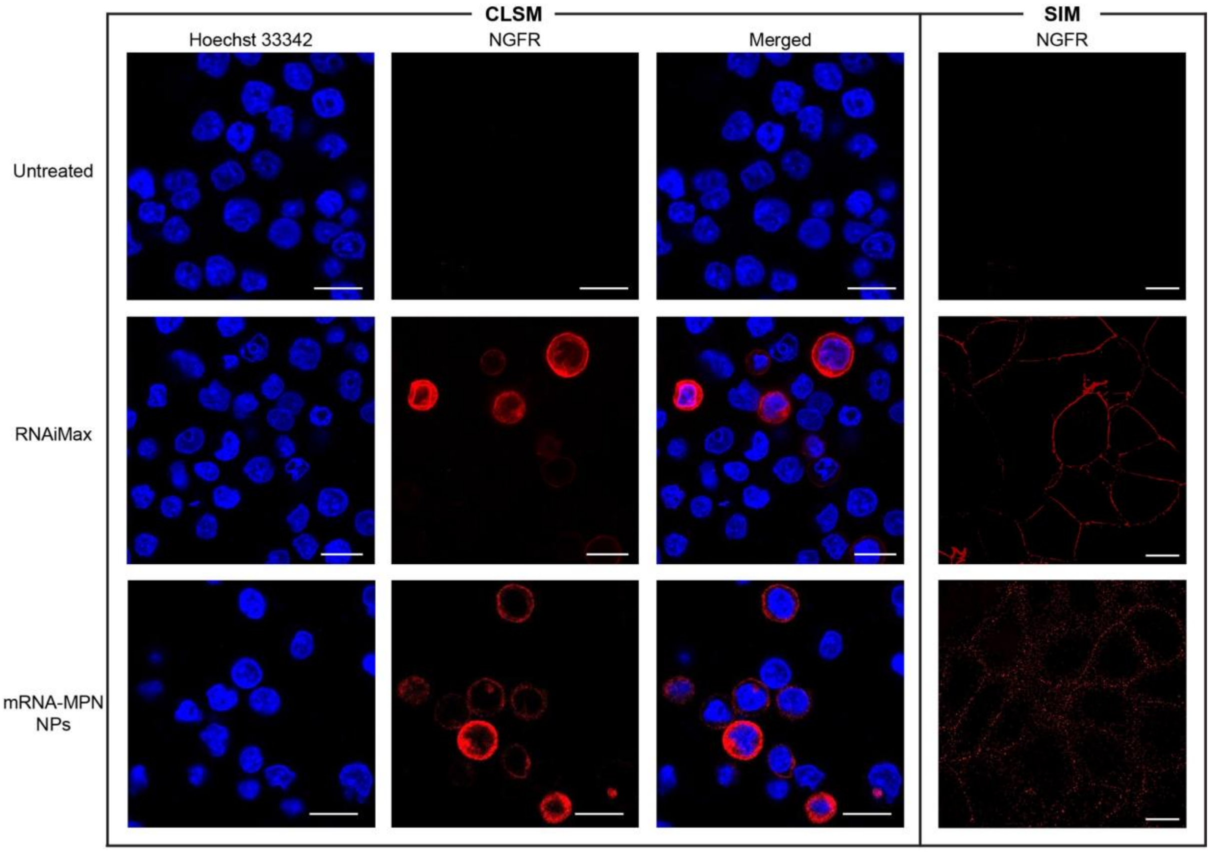


**Supplementary Fig. 21|** Representative CLSM and Lattice-SIM images showing the transfection of surface-expressed NGFR in HEK 293T cells after 24 h. NGFR was stained with phycoerythrin-conjugated anti-NGFR. Scale bars are 10 µm. Experiments were performed in triplicates.

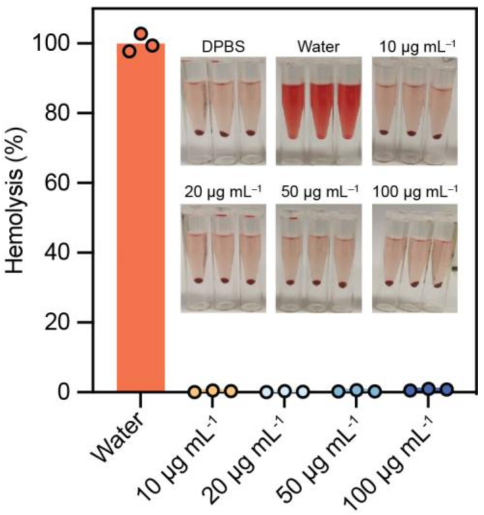


**Supplementary Fig. 22 |** Hemolysis percentage of RBCs treated with mRNA-MPN NPs at different concentrations for 2 h. RBC treated with water was set as the positive control and DPBS-treated RBC was set as the negative control. All experiments were performed in triplicates (*n* = 3) and data are presented as the mean ± SD. Source data are provided as a Source Data file.

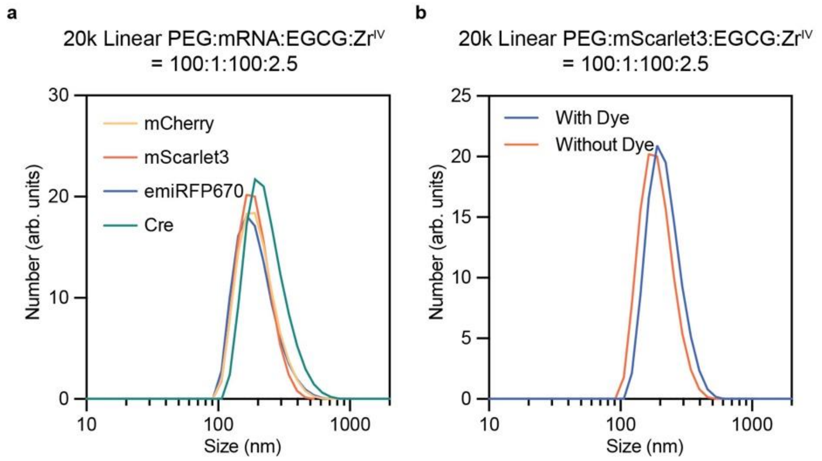


**Supplementary Fig. 23 | a,b,** Size comparisons of mRNA-MPN NPs (lead formulation) assembled with different mRNA sequences (**a**) and modification (**b**). The fluorescence dye was mixed in the formulation at an EGCG-to-dye mass ratio of 5:1. Altering the type of mRNA or adding a fluorescence dye to mRNA-MPN NPs only minimally changed the overall particle size. Source data are provided as a Source Data file.

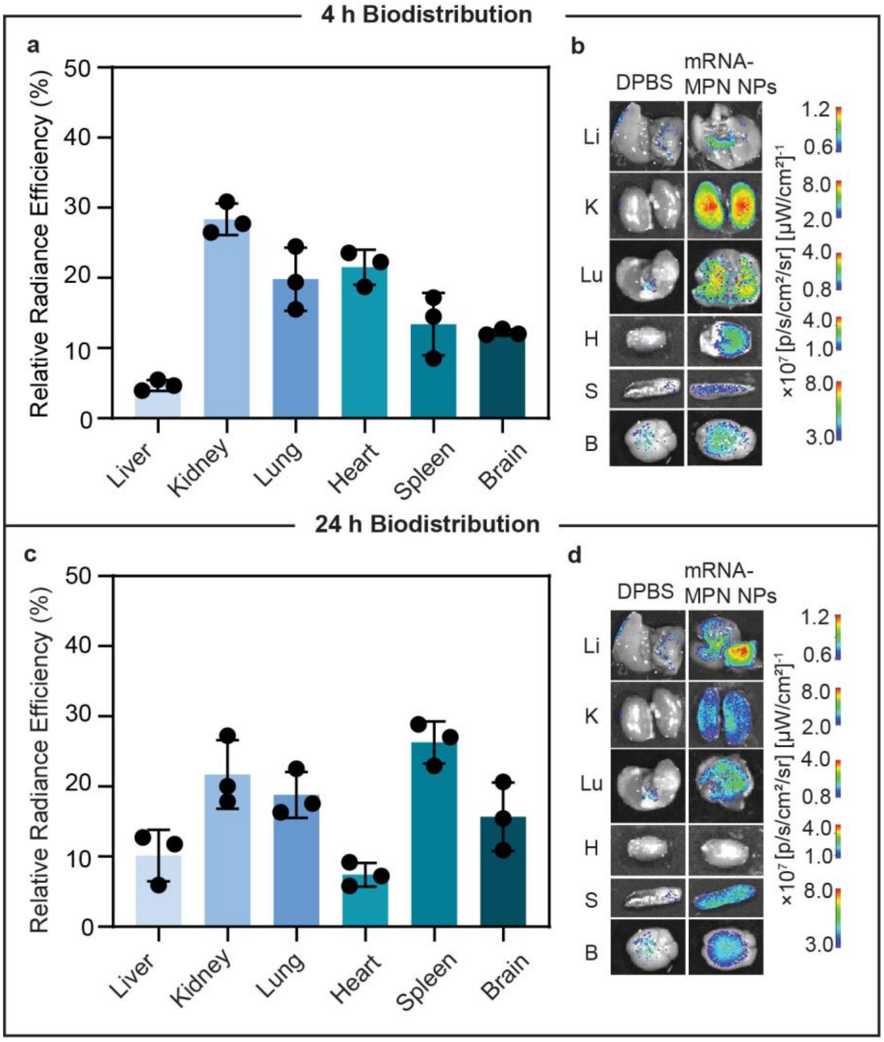


**Supplementary Fig. 24 | a-d,** Biodistribution of fluorescence (Rh800)-labeled mRNA-MPN NPs 4 h (**a**,**b**) and 24 h (**c**,**d**) after IV administration into C57BL/6J mice. Both quantitative results (bar diagrams) and inset representative images were obtained by IVIS. For all quantitative data, three biologically independent mice were included in each group ($n$ = 3), and the quantitative data were normalized to DPBS (negative control) and presented as the mean ± SD. Li, liver; K, kidney; Lu, lung; H, heart; S, spleen; B, brain. Source data are provided as a Source Data file.

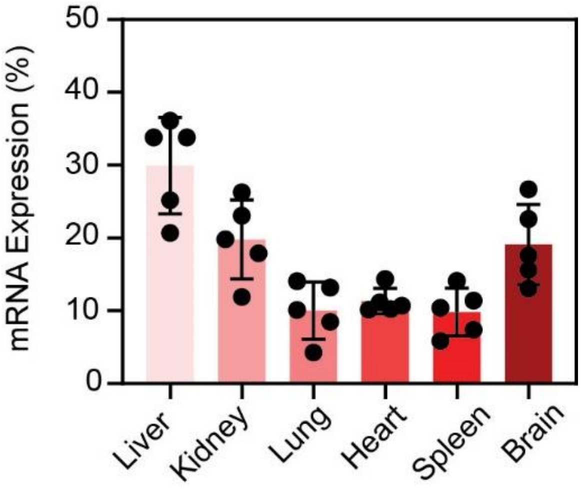


**Supplementary Fig. 25 |** Percentage of mScarlet3 expression in harvested organs. Five biologically independent mice were included in each group ($n$ = 5), and the quantitative data were normalized to DPBS (negative control) and presented as the mean ± SD. Source data are provided as a Source Data file.

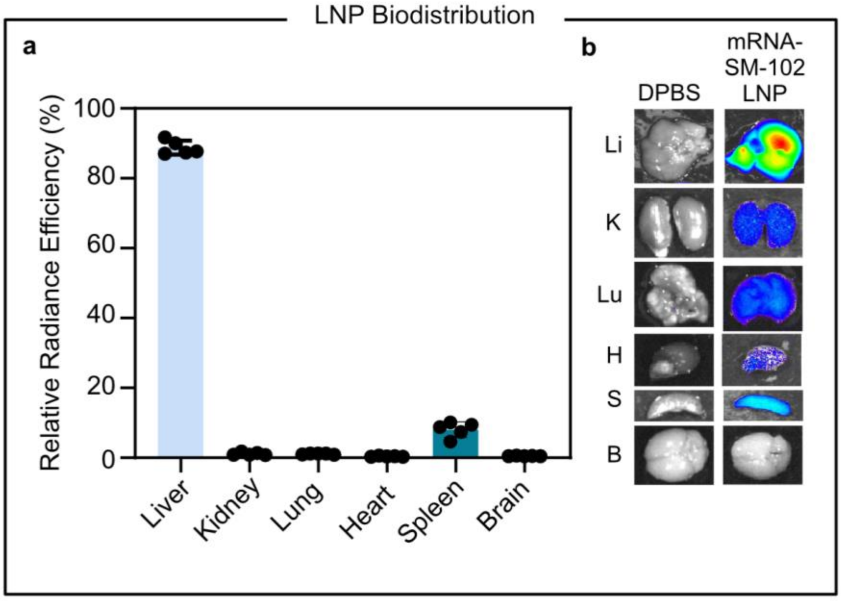


**Supplementary Fig. 26 | a,b**, Biodistribution of fluorescence (DiR)-labeled mRNA-SM-102 LNPs in different organs: both quantitative (**a**) and representative images (**b**) were obtained by IVIS 24 h post injection with an mRNA dose of 0.25 mg $kg^{-1}$. Five biologically independent mice were included in each group ($n = 5$), and the quantitative data were normalized to DPBS (negative control) and presented as the mean ± SD. Source data are provided as a Source Data file.

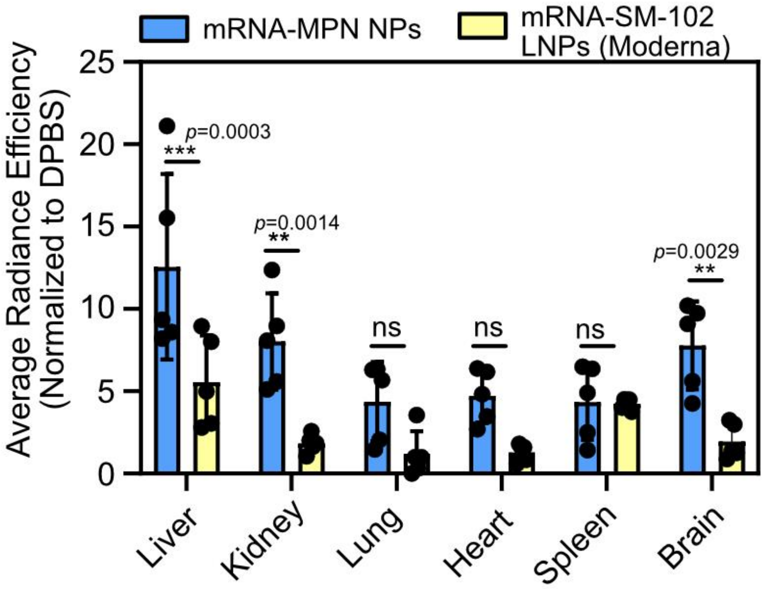


**Supplementary Fig. 27 |** mScarlet3 expression in different organs of C57BL/6J mice analyzed by IVIS 24 h post IV injection of mRNA-MPN NPs (lead formulation) and mRNA-SM-102 LNPs (Moderna formulation); mRNA dosage is 0.25 mg $kg^{-1}$. Five biologically independent mice were included in each group ($n = 5$), and the quantitative data were normalized to DPBS (negative control) and presented as the mean ± SD. Statistical significance was analyzed using two-way ANOVA with Šídák's multiple comparisons test: ns, $p$(Lung) = 0.2446; $p$(Heart) = 0.1815; $p$(Spleen) > 0.9999; ***(Liver), $p = 0.0003$; **(Kidney), $p = 0.0014$; **(Brain), $p = 0.0029$. Source data are provided as a Source Data file.

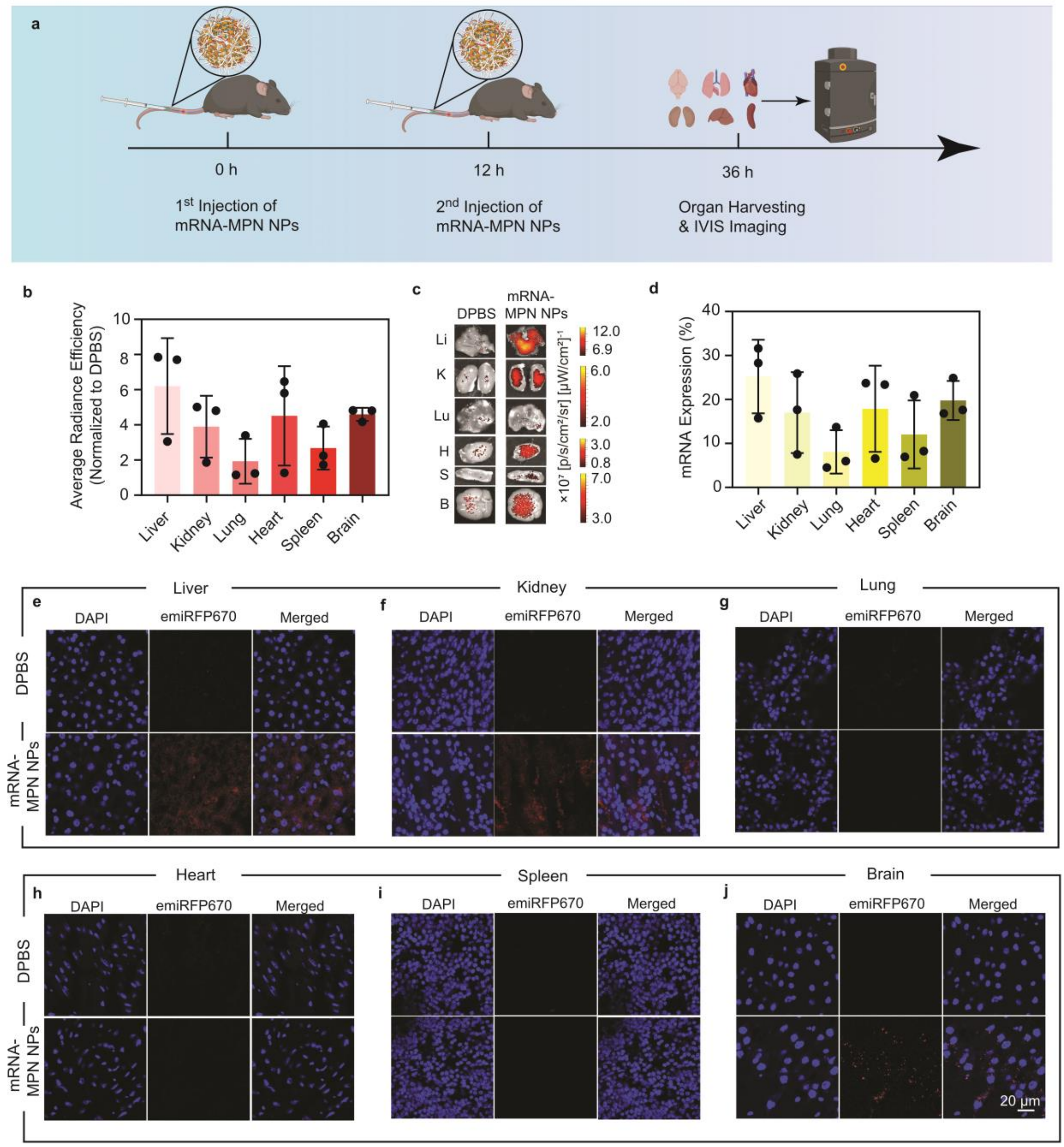


**Supplementary Fig. 28 |** In vivo emiRFP670 transfection in C57BL/6J mice using mRNA-MPN NPs. **a,** Schematic illustration of IV administration of emiRFP670-MPN NPs and subsequent organ harvesting and IVIS imaging. **b,c,** Quantitative (by IVIS) (**b**) and qualitative (representative IVIS images) (**c**) analyses of emiRFP670 transfection in harvested organs. **d,** Percentage of emiRFP670 expression in harvested organ. Three biologically independent mice were included in each group (*n* = 3), and the quantitative data were normalized to DPBS (negative control) and presented as the mean ± SD. **e-j,** CLSM images of sectioned organs of mice treated by DPBS (negative control) and emiRFP670-MPN NPs. Li, liver; K, kidney; Lu, lung; H, heart; S, spleen; B, brain. Panel (**a**) was created with BioRender.com. Source data are provided as a Source Data file.

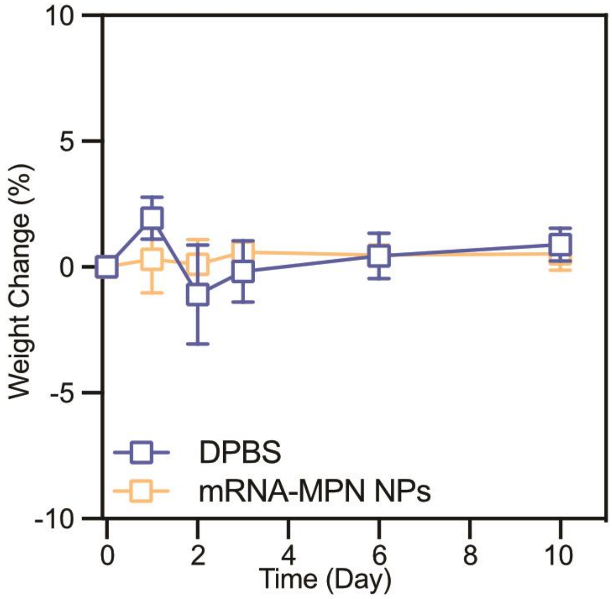


**Supplementary Fig. 29 |** Change in mouse body weight over 10 days after NP administration. Three biologically independent mice were included in each group (*n* = 3) and presented as the mean ± SD. Source data are provided as a Source Data file.

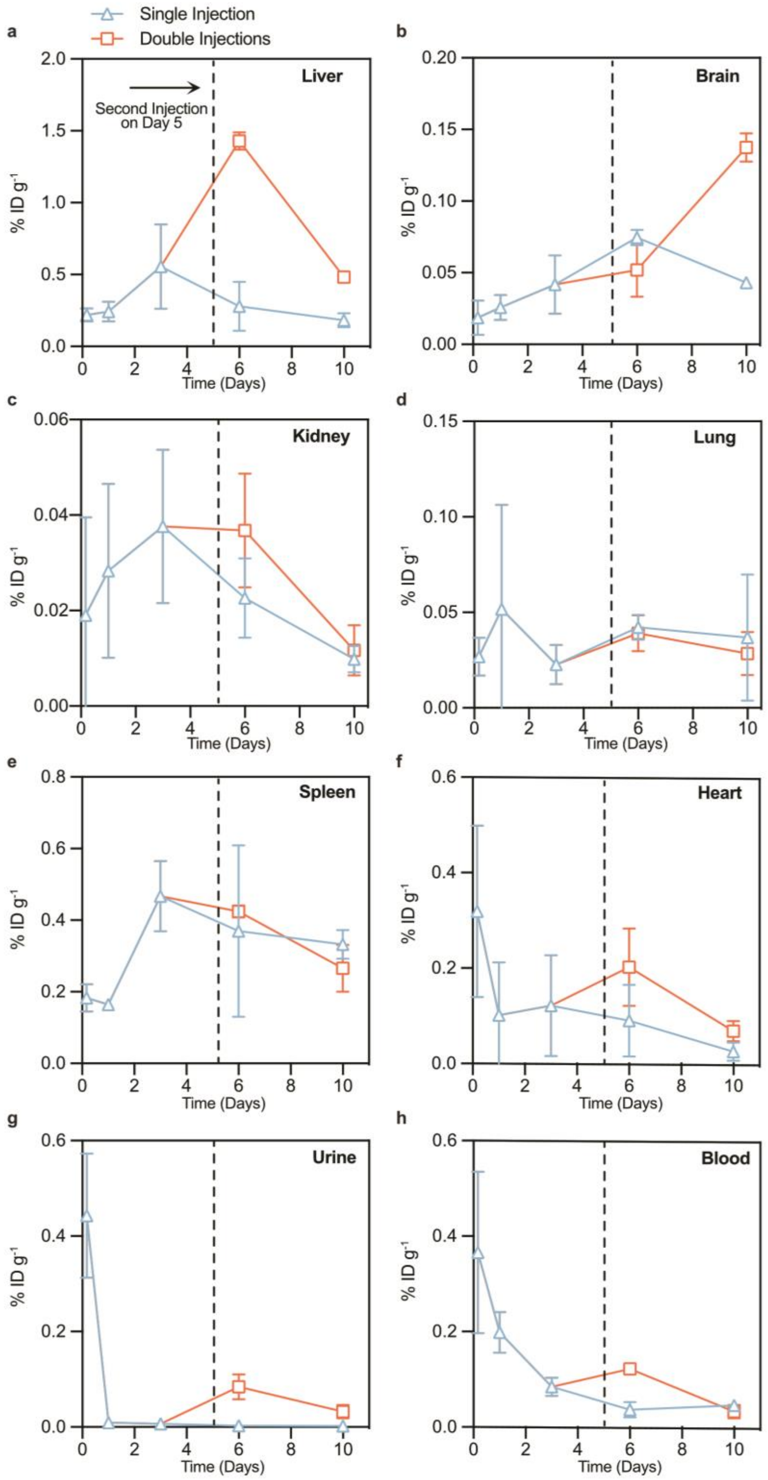


**Supplementary Fig. 30 | a–h**, Zr excretion profile in the liver (**a**), brain (**b**), kidney (**c**), lung (**d**), spleen (**e**), heart (**f**), urine (**g**), and blood (**h**) corresponding to single and double administration of mRNA-MPN NPs. Zr content in harvested organs, urine, and blood is presented as % ID $g^{-1}$. Note the Zr content in the brain after double administration did not decrease at day 10. The Zr signal peaked on day 6 after the first injection, thus the peak after the second injection was likely to occur at day 10 or day 11. However, the overall % ID $g^{-1}$ of Zr in the brain is <0.2%. Therefore, this result did not change our conclusions. Three biologically independent mice were included in each group ($n$ = 3) and presented as the mean ± SD. Source data are provided as a Source Data file.

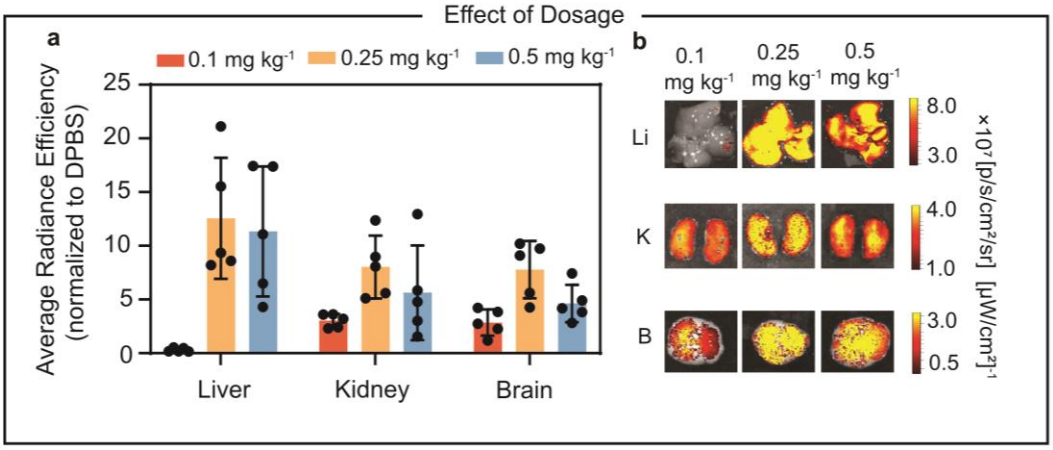


**Supplementary Fig. 31 |** In vivo mScarlet3 expression in harvested organs of mRNA-MPN NP-treated C57BL/6J mice at various mScarlet3 dosages. Both quantitative results (bar diagram) (**a**) and representative images (**b**) were obtained by IVIS. Five biologically independent mice were included in each group ($n = 5$), and the quantitative data were normalized to DPBS (negative control) and presented as mean ± SD. Li, liver; K, kidney; B, brain. Source data are provided as a Source Data file.

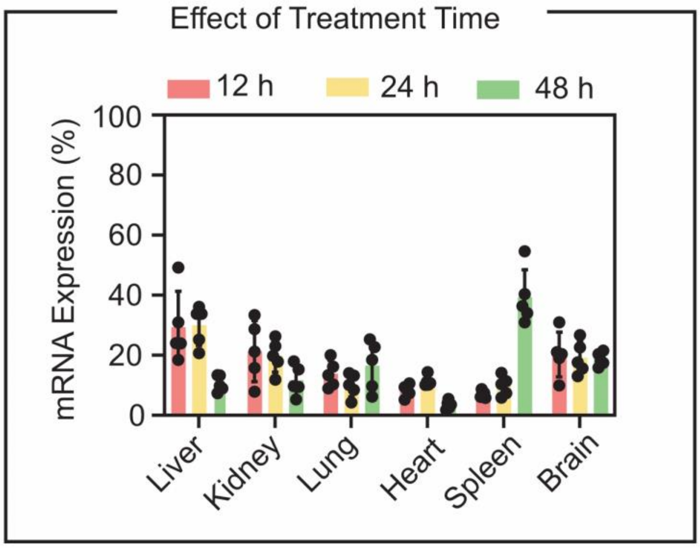


**Supplementary Fig. 32 |** Percentage of mScarlet3 expression in organs harvested from C57BL/6J mice at 12, 24, and 48 h post treatment. Five biologically independent mice were included in each group (*n* = 5), and the data (obtained from IVIS) were normalized to DPBS (negative control) and presented as mean ± SD. Source data are provided as a Source Data file.

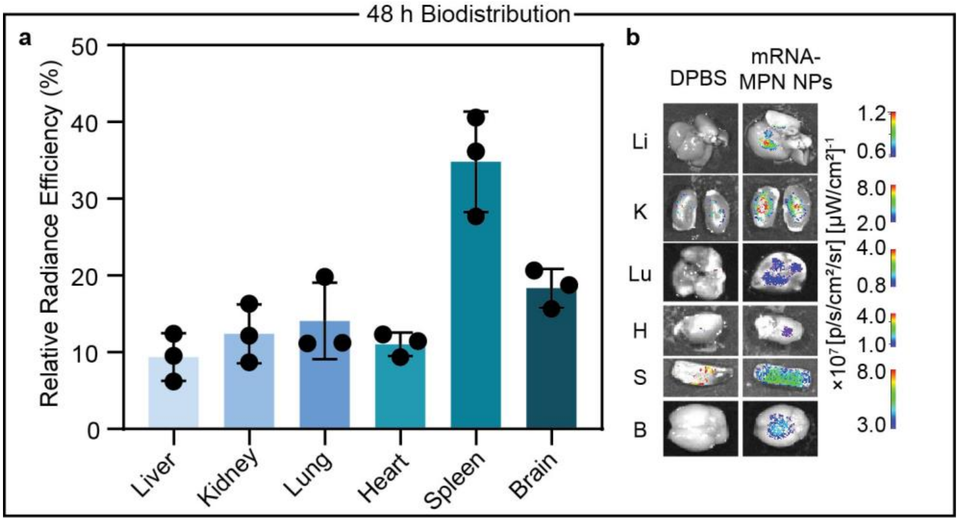


**Supplementary Fig. 33 |** Biodistribution of fluorescence (Rh800)-labeled mRNA-MPN NPs 48 h after IV administration into C57BL/6J mice. Both quantitative results (bar diagram) (**a**) and representative images (**b**) were obtained by IVIS. Three biologically independent mice were included in each group (*n* = 3), and the quantitative data were normalized to DPBS (negative control) and presented as mean ± SD. Li, liver; K, kidney; Lu, lung; H, heart; S, spleen; B, brain. Source data are provided as a Source Data file.

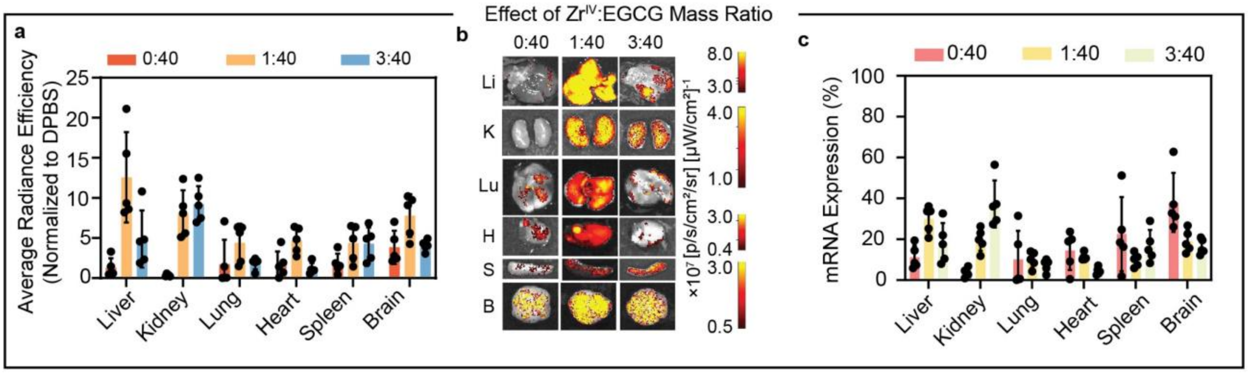


**Supplementary Fig. 34 |** Effect of Zr$^{IV}$-to-EGCG mass ratio on in vivo mScarlet3 transfection in C57BL/6J mice. **a,b,** Fluorescence intensity (**a**) and representative images (**b**) of expressed mScarlet3 in harvested organs. **c,** Percentage of mScarlet3 expression in harvested organs. Both quantitative results (bar diagrams) and inset representative images were obtained by IVIS. Five biologically independent mice were included in each group ($n = 5$), and the data were normalized to DPBS (negative control) and presented as the mean ± SD. Li, liver; K, kidney; Lu, lung; H, heart; S, spleen; B, brain. Source data are provided as a Source Data file.

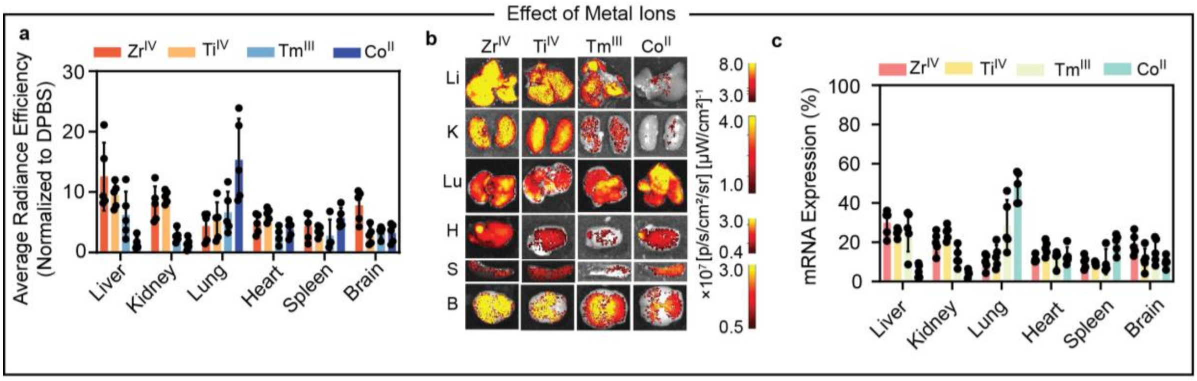


**Supplementary Fig. 35 |** Effect of metal ions on in vivo mScarlet3 transfection in C57BL/6J mice. **a,b,** Fluorescence intensity (**a**) and representative images (**b**) of mScarlet3 expression in harvested organs. **c,** Percentage of mScarlet3 expression in harvested organs. Both quantitative results (bar diagrams) and inset representative images were obtained by IVIS. Four or five biologically independent mice were included in each group ($n$ = 4 or 5), and the data were normalized to DPBS (negative control) and presented as the mean ± SD. Li, liver; K, kidney; Lu, lung; H, heart; S, spleen; B, brain. Source data are provided as a Source Data file.

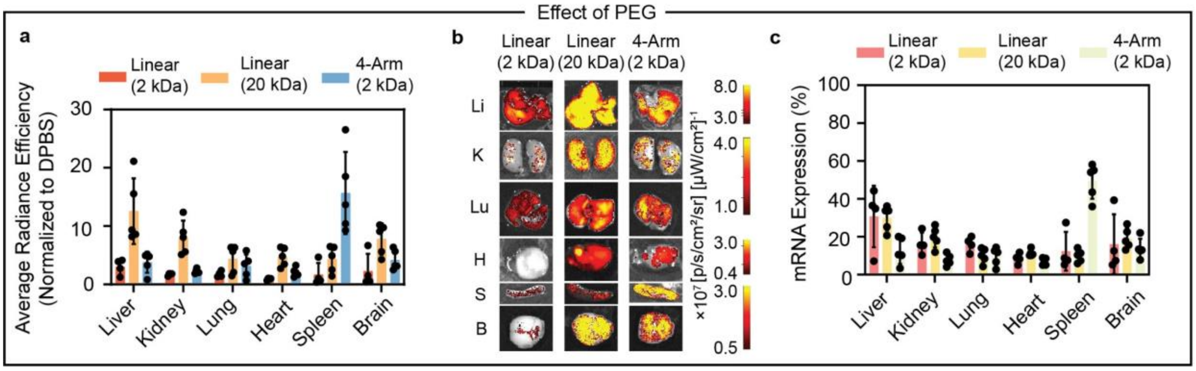


**Supplementary Fig. 36 |** Effect of PEG ($M_w$ or structure) on in vivo mScarlet3 transfection in C57BL/6J mice. **a,b,** Fluorescence intensity (**a**) and representative images (**b**) of mScarlet3 expression in harvested organs. **c,** Percentage of mScarlet3 expression in harvested organs. Both quantitative results (bar diagrams) and inset representative images were obtained by IVIS. Four or five biologically independent mice were included in each group (*n* = 4 or 5), and the data were normalized to DPBS (negative control) and presented as the mean ± SD. Li, liver; K, kidney; Lu, lung; H, heart; S, spleen; B, brain. Source data are provided as a Source Data file.

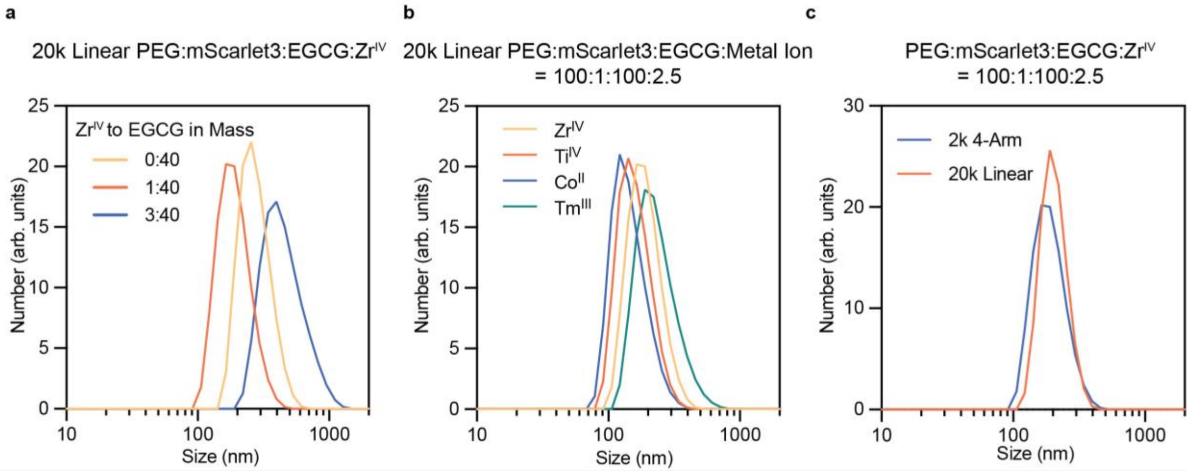


**Supplementary Fig. 37 | a-c,** Size comparison of selected high-performance mRNA-MPN NP formulations used for in vivo studies differing in $Zr^{IV}$-to-EGCG mass ratio (**a**), metal ions (**b**), or PEG ($M_w$ or structure) (**c**). Source data are provided as a Source Data file.

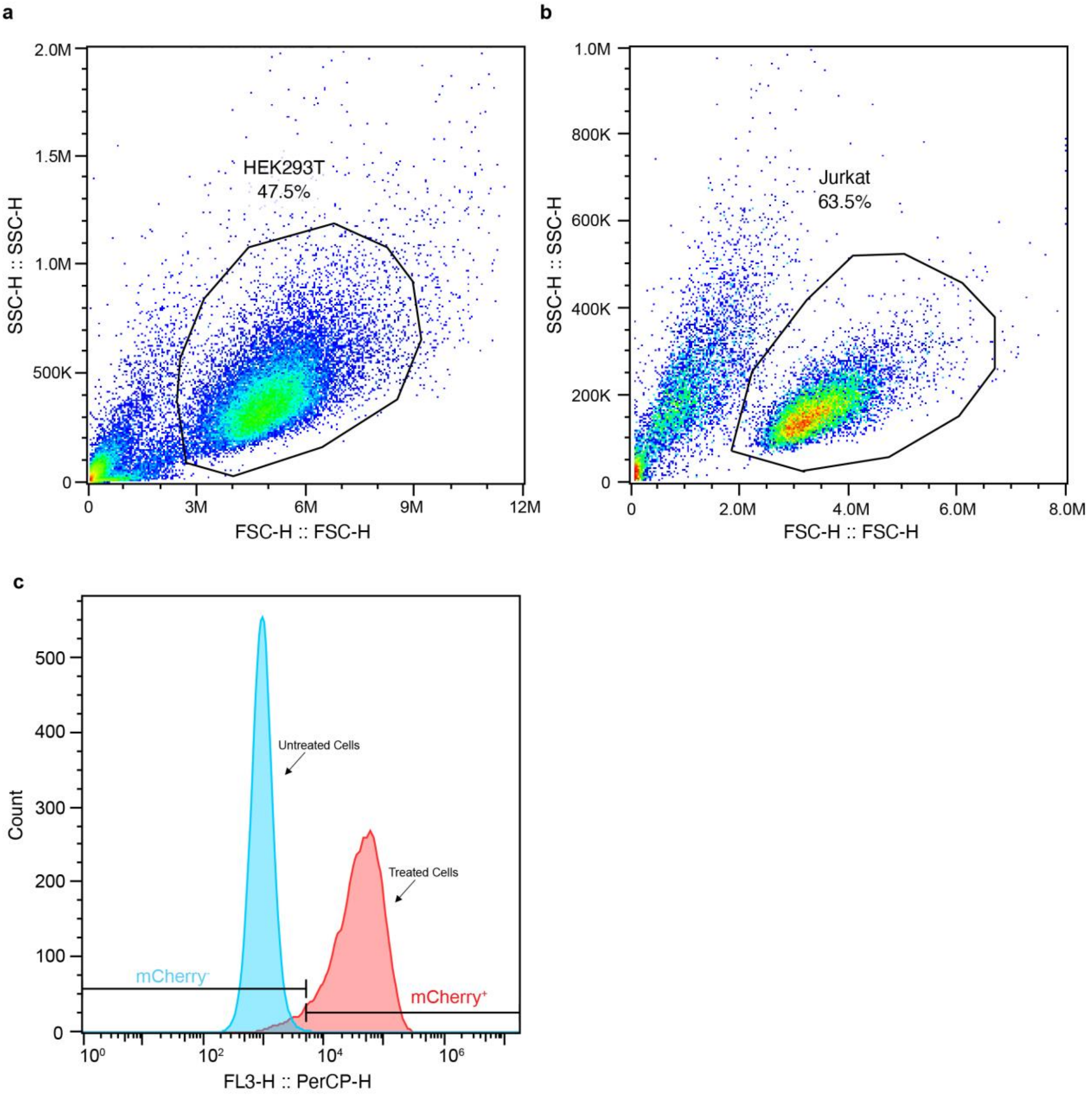


**Supplementary Fig. 38 | a-c,** Representative gating strategies used for flow cytometry for HEK 293T cells (**a**), Jurkat cells (**b**), and determination of mCherry$^+$ population (**c**).

## Supplementary Table 1 | Characterization of mRNA-MPN NPs (lead formulation) with different mRNA sequences[a]

| Metal Ion | PEG | PEG:mRNA:EGCG:Metal Ion Mass Ratio | Diameter (nm) | PDI[b] | ζ-Potential (mV)[c] |
|---|---|---|---|---|---|
| $Zr^{IV}$ | 20k Linear | 100:1(mCherry):100:2.5 | 223 ± 26 | 0.19 | −18.3 ± 3.6 |
| $Zr^{IV}$ | 20k Linear | 100:1(mScarlet3):100:2.5 | 220 ± 37 | 0.05 | −17.2 ± 4.3 |
| $Zr^{IV}$ | 20k Linear | 100:1(emiRFP670):100:2.5 | 203 ± 40 | 0.19 | −18.1 ± 2.7 |
| $Zr^{IV}$ | 20k Linear | 100:1(Cre):100:2.5 | 243 ± 28 | 0.30 | −22.9 ± 3.1 |

[a]Data are presented as the mean ± SD of three independently synthesized batches of NPs ($n$ = 3).
[b]Characterized by dynamic light scattering.
[c]Characterized by dynamic light scattering in diluted DPBS (10 mM, pH 7.4).

## Supplementary Table 2 | mRNA sequences

| mRNA Sequence | mRNA Length (nucleotide number) | Maximum Excitation/Emission Wavelengths of Expressed Fluorescence Protein (nm/nm)[a] |
|---|---|---|
| mCherry | 1056 | 587/610 |
| mScarlet3 | 1004 | 561/594 |
| emiRFP670 | 1232 | 642/670 |
| Cre | 1336 | - |
| FLuc | 1998 | - |
| NGFR | 1216 | - |

[a]Excitation/Emission wavelengths not applicable for mRNA-encoding non-fluorescence proteins.

## Supplementary Table 3 | Characterization of mRNA-MPN NPs prepared using the lead formulation without or with fluorescent dye labeling[a]

| Metal Ion | PEG | PEG:mScarlet3:EGCG:Metal Ion:Dye Mass Ratio | Diameter (nm) | PDI[b] | ζ-Potential (mV)[c] |
|---|---|---|---|---|---|
| $Zr^{IV}$ | 20k Linear | 100:1:100:2.5:0 | 220 ± 37 | 0.05 | −17.2 ± 4.3 |
| $Zr^{IV}$ | 20k Linear | 100:1:100:2.5:20 | 233 ± 26 | 0.11 | −17.8 ± 4.6 |

[a]Data are presented as the mean ± SD of three independently synthesized batches of NPs ($n$ = 3).
[b]Characterized by dynamic light scattering.
[c]Characterized by dynamic light scattering in diluted DPBS (10 mM, pH 7.4).

## Supplementary Table 4 | Characterization of mRNA-MPN NP formulations prepared using different $Zr^{IV}$-to-EGCG mass ratios and examined in in vivo studies[a]

| Metal Ion | PEG | PEG:mScarlet3:EGCG:Metal Ion Mass Ratio | Diameter (nm) | PDI[b] | ζ-Potential (mV)[c] |
|---|---|---|---|---|---|
| $Zr^{IV}$ | 20k Linear | 100:1:100:2.5 | 220 ± 37 | 0.05 | −17.2 ± 4.3 |
| - | 20k Linear | 100:1:100:0 | 277 ± 36 | 0.09 | −23.4 ± 4.3 |
| $Zr^{IV}$ | 20k Linear | 100:1:100:7.5 | 462 ± 47 | 0.09 | −30.5 ± 2.3 |

[a]Data are presented as the mean ± SD of three independently synthesized batches of NPs ($n$ = 3).
[b]Characterized by dynamic light scattering.
[c]Characterized by dynamic light scattering in diluted DPBS (10 mM, pH 7.4).

## Supplementary Table 5 | Characterization of mRNA-MPN NP (lead formulation) prepared with different metal ions and examined in *in vivo* studies[a]

| Metal Ion | PEG | PEG:mScarlet3:EGCG:Metal Ion Mass Ratio | Diameter (nm) | PDI[b] | ζ-Potential (mV)[c] |
|---|---|---|---|---|---|
| $Zr^{IV}$ | 20k Linear | 100:1:100:2.5 | 220 ± 37 | 0.05 | −17.2 ± 4.3 |
| $Ti^{IV}$ | 20k Linear | 100:1:100:2.5 | 162 ± 17 | 0.04 | −10.5 ± 0.7 |
| $Tm^{III}$ | 20k Linear | 100:1:100:2.5 | 240 ± 49 | 0.11 | −22.7 ± 3.1 |
| $Co^{II}$ | 20k Linear | 100:1:100:2.5 | 148 ± 13 | 0.09 | −12.6 ± 1.9 |

[a]Data are presented as the mean ± SD of three independently synthesized batches of NPs (*n* = 3).
[b]Characterized by dynamic light scattering.
[c]Characterized by dynamic light scattering in diluted DPBS (10 mM, pH 7.4).

## Supplementary Table 6 | Characterization of mRNA-MPN NPs (lead formulation) prepared by varying the $M_w$ or structure of PEG and examined in *in vivo* studies[a]

| Metal Ion | PEG | PEG:mScarlet3:EGCG:Metal Ion Mass Ratio | Diameter (nm) | PDI[b] | ζ-Potential (mV)[c] |
|---|---|---|---|---|---|
| $Zr^{IV}$ | 20k Linear | 100:1:100:2.5 | 220 ± 37 | 0.05 | −17.2 ± 4.3 |
| $Zr^{IV}$ | 2k 4-Arm | 100:1:100:2.5 | 206 ± 21 | 0.06 | −47.0 ± 5.4 |

[a]Data are presented as the mean ± SD of three independently synthesized batches of NPs (*n* = 3).
[b]Characterized by dynamic light scattering.
[c]Characterized by dynamic light scattering in diluted DPBS (10 mM, pH 7.4).

# Supplementary References


1 Branchini, B. R., Southworth, T. L., Murtiashaw, M. H., Boije, H. & Fleet, S. E. A mutagenesis study of the putative luciferin binding site residues of firefly luciferase. *Biochemistry* **42**, 10429-10436 (2003).

2 Branchini, B. R., Magyar, R. A., Murtiashaw, M. H., Anderson, S. M. & Zimmer, M. Site-directed mutagenesis of histidine 245 in firefly luciferase: a proposed model of the active site. *Biochemistry* **37**, 15311-15319 (1998).

**Checklist**

**Minimum Information Reporting in Bio–Nano Experimental Literature**

The MIRIBEL guidelines were introduced here: https://doi.org/10.1038/s41565-018-0246-4

The development of these guidelines was led by the ARC Centre of Excellence in Convergent Bio-Nano Science and Technology: https://www.cbns.org.au/. Any updates or revisions to this document will be made available here: http://doi.org/10.17605/OSF.IO/SMVTF. This document is made available under a CC-BY 4.0 license: https://creativecommons.org/licenses/by/4.0/.

The MIRIBEL guidelines were developed to facilitate reporting and dissemination of research in bio–nano science. Their development was inspired by various similar efforts:

- MIAME (microarray experiments): *Nat. Genet.* **29** (2001), 365; http://doi.org/10.1038/ng1201-365
- MIRIAM (biochemical models): *Nat. Biotechnol.* **23** (2005) 1509; http://doi.org/10.1038/nbt1156
- MIBBI (biology/biomedicine): *Nat. Biotechnol.* **26** (2008) 889; http://doi.org/10.1038/nbt.1411
- MIGS (genome sequencing): *Nat. Biotechnol.* **26** (2008) 541; http://doi.org/10.1038/nbt1360
- MIQE (quantitative PCR): *Clin. Chem.* **55** (2009) 611; http://doi.org/10.1373/clinchem.2008.112797
- ARRIVE (animal research): *PLOS Biol.* **8** (2010) e1000412; http://doi.org/10.1371/journal.pbio.1000412
- *Nature*'s reporting standards:
    - Life science: https://www.nature.com/authors/policies/reporting.pdf; e.g., *Nat. Nanotechnol.* **9** (2014) 949; http://doi.org/10.1038/nnano.2014.287
    - Solar cells: https://www.nature.com/authors/policies/solarchecklist.pdf; e.g., *Nat. Photonics* **9** (2015) 703; http://doi.org/10.1038/nphoton.2015.233
    - Lasers: https://www.nature.com/authors/policies/laserchecklist.pdf; e.g., *Nat. Photonics* **11** (2017) 139; http://doi.org/10.1038/nphoton.2017.28
- The "TOP guidelines": e.g., *Science* **352** (2016) 1147; http://doi.org/10.1126/science.aag2359

Similar to many of the efforts listed above, the parameters included in this checklist are **not** intended to be definitive requirements; instead they are intended as 'points to be considered', with authors themselves deciding which parameters are—and which are not—appropriate for their specific study.

This document is intended to be a living document, which we propose is revisited and amended annually by interested members of the community, who are encouraged to contact the authors of this document. Parts of this document were developed at the annual International Nanomedicine Conference in Sydney, Australia: http://www.oznanomed.org/, which will continue to act as a venue for their review and development, and interested members of the community are encouraged to attend.

After filling out the following pages, this checklist document can be attached as a "Supporting Information" document during submission of a manuscript to inform Editors and Reviewers (and eventually readers) that all points of MIRIBEL have been considered.

**Supplementary Table 1. Material characterization***

| Question | Yes | No |
|---|---|---|
| 1.1 Are "**best reporting practices**" **available** for the nanomaterial used? For examples, see *Chem. Mater.* **28** (2016) 3535; http://doi.org/10.1021/acs.chemmater.6b01854 and *Chem. Mater.* **29** (2017) 1; http://doi.org/10.1021/acs.chemmater.6b05235 | Not applicable | |
| 1.2 If they are available, **are they used**? If not available, ignore this question and proceed to the next one. | | |
| 1.3 Are extensive and clear instructions reported detailing all steps of **synthesis** and the resulting **composition** of the nanomaterial? For examples, see *Chem. Mater.* **26** (2014) 1765; http://doi.org/10.1021/cm500632c, and *Chem. Mater.* **26** (2014) 2211; http://doi.org/10.1021/cm5010449. Extensive use of photos, images, and videos are strongly encouraged. For example, see *Chem. Mater.* **28** (2016) 8441; http://doi.org/10.1021/acs.chemmater.6b04639 | ✓ | |
| 1.4 Is the **size** (or **dimensions**, if non-spherical) and **shape of** the nanomaterial reported? | ✓ | |
| 1.5 Is the **size dispersity** or **aggregation** of the nanomaterial reported? | ✓ | |
| 1.6 Is the **zeta potential** of the nanomaterial reported? | ✓ | |
| 1.7 Is the **density (mass/volume)** of the nanomaterial reported? | Not applicable | |
| 1.8 Is the amount of any **drug loaded** reported? 'Drug' here broadly refers to functional cargos (e.g., proteins, small molecules, nucleic acids). | ✓ | |
| 1.9 Is the **targeting performance** of the nanomaterial reported, including **amount** of ligand bound to the nanomaterial if the material has been functionalized through addition of targeting ligands? | Not applicable | |
| 1.10 Is the **label signal** per nanomaterial/particle reported? For example, fluorescence signal per particle for fluorescently labelled nanomaterials. | | ✓ |
| 1.11 If a material property not listed here is varied, has it been **quantified**? | Not applicable | |
| 1.12 Were characterizations performed in a **fluid mimicking biological conditions**? | | ✓ |
| 1.13 Are details of how these parameters were **measured/estimated** provided? | ✓ | |
| Explanation for **No** (if needed):<br>1.10: Fluorescence labeling of mRNA-MPN NPs was achieved through the incorporation of Cy5-labeled mRNA or fluorophores (commercially available), which was characterized by fluorescence spectroscopy and Lattice-SIM.<br>1.12: ζ-Potential, intensity particle size distribution, and PDI were measured by DLS. Thus, they could not be measured in biomimetic conditions (e.g., culture media). | | |

*Ideally, material characterization should be performed in the same biological environment as that in which the study will be conducted. For example, for cell culture studies with nanoparticles, characterization steps would ideally be performed on nanoparticles dispersed in cell culture media. If this is not possible, then characteristics of the dispersant used (e.g., pH, ionic strength) should mimic as much as possible the biological environment being studied.

**Supplementary Table 2. Biological characterization***

| Question | Yes | No |
|---|---|---|
| 2.1 Are **cell seeding details**, including **number of cells plated, confluency at start of experiment,** and **time between seeding and experiment** reported? | ✓ | |
| 2.2 If a standardised cell line is used, are the **designation and source** provided? | ✓ | |
| 2.3 Is the **passage number** (total number of times a cell culture has been subcultured) known and reported? | ✓ | |
| 2.4 Is the last instance of **verification of cell line** reported? If no verification has been performed, is the time passed and passage number since acquisition from trusted source (e.g., ATCC or ECACC) reported? For information, see *Science* **347** (2015) 938; http://doi.org/10.1126/science.347.6225.938 | | ✓ |
| 2.5 Are the results from **mycoplasma testing** of cell cultures reported? | ✓ | |
| 2.6 Is the **background signal of cells/tissue** reported? (E.g., the fluorescence signal of cells without particles in the case of a flow cytometry experiment.) | ✓ | |
| 2.7 Are **toxicity studies** provided to demonstrate that the material has the expected toxicity, and that the experimental protocol followed does not? | ✓ | |
| 2.8 Are details of media preparation (**type of media, serum,** any **added antibiotics**) provided? | ✓ | |
| 2.9 Is a **justification of the biological model** used provided? For examples for cancer models, see *Cancer Res.* **75** (2015) 4016; http://doi.org/10.1158/0008-5472.CAN-15-1558, and *Mol. Ther.* **20** (2012) 882; http://doi.org/10.1038/mt.2012.73, and *ACS Nano* **11** (2017) 9594; http://doi.org/10.1021/acsnano.7b04855 | Not applicable | |
| 2.10 Is characterization of the **biological fluid** (*ex vivo*/*in vitro*) reported? For example, when investigating protein adsorption onto nanoparticles dispersed in blood serum, pertinent aspects of the blood serum should be characterised (e.g., protein concentrations and differences between donors used in study). | Not applicable | |
| 2.11 For **animal experiments**, are the ARRIVE guidelines followed? For details, see *PLOS Biol.* **8** (2010) e1000412; http://doi.org/10.1371/journal.pbio.1000412 | ✓ | |
| Explanation for **No** (if needed):<br>2.4: Cells were purchased from the American Type Culture Collection. The passage number is known and standard mycoplasma test was conducted. | | |

*For *in vitro* experiments (e.g., cell culture), *ex vivo* experiments (e.g., in blood samples), and *in vivo* experiments (e.g., animal models). The questions above that are appropriate depend on the type of experiment conducted.

**Supplementary Table 3. Experimental details***

| Question | Yes | No |
|---|---|---|
| 3.1 For cell culture experiments: are **cell culture dimensions** including **type of well**, **volume of added media**, reported? Are cell types (i.e.; adherent vs suspension) and **orientation** (if non-standard) reported? | ✓ | |
| 3.2 Is the **dose of material administered** reported? This is typically provided in nanomaterial mass, volume, number, or surface area added. Is sufficient information reported so that regardless of which one is provided, the other dosage metrics can be calculated (i.e. using the dimensions and density of the nanomaterial)? | ✓ | |
| 3.3 For each type of imaging performed, are details of how **imaging** was performed provided, including details of **shielding, non-uniform image processing**, and any **contrast agents** added? | Not applicable | |
| 3.4 Are details of how the dose was administered provided, including **method of administration, injection location, rate of administration,** and details of **multiple injections**? | ✓ | |
| 3.5 Is the methodology used to **equalise dosage** provided? | ✓ | |
| 3.6 Is the **delivered dose** to tissues and/or organs (in vivo) reported, as % injected dose per gram of tissue (%ID $g^{-1}$)? | | ✓ |
| 3.7 Is **mass of each organ/tissue measured** and **mass of material** reported? | | ✓ |
| 3.8 Are the **signals of cells/tissues with nanomaterials** reported? For instance, for fluorescently labelled nanoparticles, the total number of particles per cell or the fluorescence intensity of particles + cells, at each assessed timepoint. | ✓ | |
| 3.9 Are **data analysis details**, including **code used** for analysis provided? | ✓ | |
| 3.10 Is the **raw data** or **distribution of values** underlying the reported results provided? For examples, see *R. Soc. Open Sci.* **3** (2016) 150547; http://doi.org/10.1098/rsos.150547, https://opennessinitiative.org/making-your-data-public/, http://journals.plos.org/plosone/s/data-availability, and https://www.nature.com/sdata/policies/repositories | | ✓ |
| Explanation for **No** (if needed): 3.6 and 3.7: The injection dose of mRNA-MPN NPs was determined based on the mRNA loading. The delivered dose and the mass of each organ were not measured as the injected NPs did not contain toxic compositions, and we were only interested in the organ deposition of these NPs. | | |

* The use of protocol repositories (e.g., Protocol Exchange http://www.nature.com/protocolexchange/) and published standard methods and protocols (e.g., Chem. Mater. 29 (2017) 1; http://doi.org/10.1021/acs.chemmater.6b05235, and Chem. Mater. 29 (2017) 475; http://doi.org/10.1021/acs.chemmater.6b05481) are encouraged.
